# ESIPT-Related Origin of Dual Fluorescence in the Selected Model 1,3,4-Thiadiazole Derivatives

**DOI:** 10.3390/molecules25184168

**Published:** 2020-09-11

**Authors:** Grzegorz Czernel, Iwona Budziak, Anna Oniszczuk, Dariusz Karcz, Katarzyna Pustuła, Andrzej Górecki, Alicja Matwijczuk, Bożena Gładyszewska, Mariusz Gagoś, Andrzej Niewiadomy, Arkadiusz Matwijczuk

**Affiliations:** 1 Department of Biophysics, University of Life Sciences in Lublin, Akademicka 13, 20-950 Lublin, Poland; grzegorz.czernel@up.lublin.pl (G.C.); alicja.matwijczuk@up.lublin.pl (A.M.); bozena.gladyszewska@up.lublin.pl (B.G.); 2 Department of Chemistry, University of Life Sciences in Lublin, Akademicka 15, 20-950 Lublin, Poland; iwona.budziak@up.lublin.pl; 3 Department of Inorganic Chemistry, Medical University in Lublin, 20-059 Lublin, Poland; 4 Department of Analytical Chemistry (C1), Faculty of Chemical Engineering and Technology, Cracow University of Technology, Warszawska 24, 31-155 Cracow, Poland; 5 Department of Theoretical Chemistry, Faculty of Chemistry, Jagiellonian University, Gronostajowa 2, 30-387 Kraków, Poland; pustulak@chemia.uj.edu.pl; 6 Department of Physical Biochemistry, Faculty of Biochemistry, Biophysics and Biotechnology of the Jagiellonian University, Gronostajowa 7, 30-387 Kraków, Poland; andrzej.gorecki@uj.edu.pl; 7 Department of Cell Biology, Institute of Biology and Biochemistry, Maria Curie-Skłodowska University, Akademicka 19, 20-033 Lublin, Poland; mariusz.gagos@poczta.umcs.lublin.pl; 8 Institute of Industrial Organic Chemistry, Annopol 6, 03-236 Warsaw, Poland; andrzej.niewiadomy@up.lublin.pl

**Keywords:** dual fluorescence effect, ESIPT and AIE, new derivative of 1,3,4-thiadiazoles, keto tautomer in excited state

## Abstract

In our previous work, we discussed the emergence of the dual fluorescence phenomenon in selected compounds from the group of 1,3,4-thiadiazoles. The results obtained in a number of experimental studies, supported by [TD]DFT calculations, clearly indicated that the phenomenon of dual fluorescence stemmed from an overlap of several factors, including the correct conformation of the analyzed molecule and, very significantly in this context, aggregation effects. Where those two conditions were met, we could observe the phenomenon of intermolecular charge transfer (CT) and the emergence of electronic states responsible for long wave emissions. However, in light of the new studies presented in this paper, we were able, for the first time, to provide a specific theory for the effect of dual fluorescence observed in the analyzed group of 1,3,4-thiadiazoles. We present the results of spectroscopic measurements conducted for two selected analogues from the 1,3,4-thiadiazole group, both in polar and non-polar solvents, which clearly evidence (as we have already suspected in the past, albeit have not shown in publications to date) the possibility of processes related to emission from the tautomer formed in the process of excited state intramolecular proton transfer, which is responsible for the long-wavelength emissions observed in the selected analogues. The presented results obtained with the use of UV-Vis, fluorescence (stationary and time-resolved), FTIR, and Raman spectroscopy, as well as from calculations of dipole moment changes between the ground and excited state with the use of two derivatives with different structures of the resorcylic system, corroborated our standing hypothesis. At the same time, they excluded the presence of ground state keto forms of the analyzed analogues unless necessitated by the structure of the molecule itself. In this case, aggregation factors enhance the observed effects related to the dual fluorescence of the analyzed compounds (by way of AIE—aggregated induced emissions).

## 1. Introduction

The significance of compounds from the 2-amino-1,3,4-thiadiazole group [1,2,3,4,5] stems from their potential value in the fight against some of the most challenging diseases of the 21st century, including, e.g., cancer, neurodegenerative diseases, or various continuously mutating mycoses. These compounds show a wide spectrum of pharmacological properties, including neuroprotective [6,7], anti-cancer [8], antibacterial [9], antioxidative [10,11], and antimycotic [12,13] effects. Contemporary researchers working in the fields of biophysics and photophysics or photochemistry and utilizing the methods of molecular spectroscopy are faced with the extremely difficult task, posed by medical and pharmaceutical sciences, of determining the molecular properties of continuously developed new compounds [1,2,14,15,16,17,18].

**Scheme 1:**
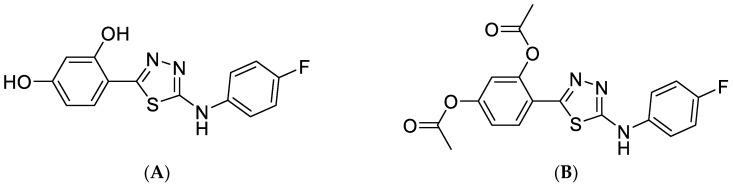
The chemical structure of compounds: (**A**) 2-((4-fluorophenyl)amino)-5-(2,4-dihydroxybenzeno)-1,3,4-thiadiazole (FABT); (**B**) 2-acetamido-5-((phenyl-2,4-diacetate)-yl)-1,3,4-thiadiazole (ADAPT).

The following compounds were selected for the present study on the mechanisms of molecular interactions in various types of solvents: 2-((4-fluorophenyl)amino)-5-(2,4-dihydroxybenzeno)-1,3,4-thiadiazole (FABT) and 2-acetamido-5-((phenyl-2,4-diacetate)-yl)-1,3,4-thiadiazole (ADAPT) (Scheme 1A,B). It should be noted that the selected analogues are characterized by not only very promising biological effects but also very interesting spectroscopic and crystallographic properties. The same include effects related to crystal polymorphism and solvatomorphism [19], impact on the dynamics of model biological membranes [20], changing fluorescence properties in micellar systems [1], as well as metal-chelating ability [21]. It is evident that the ability to correlate said spectroscopic and structural properties of the analyzed molecules may play a very important part in explaining the phenomena responsible for their pharmacological effects. Moreover, the 1,3,4-thiadiazole analogues selected for this study additionally show a very interesting effect of dual fluorescence emission [22,23], which in this particular case, based on our studies conducted to date, may be induced by a number of factors, specifically: changes in the polarity [14], pH [4,18,24], temperature [4] or the concentration of the compound itself [4].

As for the effect of dual fluorescence [25] as such, the literature provides several explanations based on the most popular theories, including that of intermolecular charge transfer (CT) inducting the emergence of the required CT states [26,27,28], which may be associated with molecule twisting producing the so-called TICT effect (twisted intramolecular charge transfer) [29,30,31]. Naturally, molecules showing TICT fluorescence do not always produce dual fluorescence; there have recently been an increasing number of literature reports discussing this type of processes and systems leading to non-radiative deactivation of the excited state. Moreover, the molecules may also show the presence of multi-stage phototautomerization processes, ESIPT (excited state intramolecular proton transfer) [32,33,34,35,36]. The properties characterizing ESIPT molecules include dual fluorescence emission, a relatively significant Stokes shift, exceptional process dynamics, as well as high sensitivity of emission spectra to changing properties of the medium [37,38,39,40]. Additionally, Yamaguchi et al. reported on quite non-typical ESIPT-related dual emission in compounds stabilized in their zwitterionic form in the excited state [41,42]. Other factors include long-wavelength fluorescence originating from the formation of excimers [43] and processes related to breaking Kasha’s rule [44,45]. In this context, one should also mention aggregation-induced emission effects (AIE) which are currently increasingly discussed in the literature [43]. At present, more and more publications report on certain particularly interesting phenomena observed for a variety of molecules, where the effects of dual fluorescence are produced through a combination of, e.g., AIE and TICT/ESIPT.

In our earlier studies on various 1,3,4-thiadiazole analogues, we were able to associate the effect of dual fluorescence observed in the same with two main factors [1,2,18]. The first is the presence of a specific conformation of the molecule which must accept the conformation with the –OH group from the resorcylic ring, in the *ortho* position, on the side of the nitrogen atom [4]. The second most important factor affecting the observed fluorescence effects is aggregation, which seems to be the necessary condition for further processes associated with the dual fluorescence of those molecules [4]. Only when the two factors are present simultaneously can the effect of dual fluorescence emission occur.

However, given the availability of new results obtained over numerous years of studies focused on this particular group of compounds, we decided to take this opportunity to explain, more accurately and in far greater detail, the mechanism of the observed effects. As provided in literature dedicated to this specific phenomenon, molecules whose structure contains, in close vicinity, groups that may serve the role of a proton donor (e.g., the –OH group in the *ortho* position from the resorcylic ring) and proton acceptor (N atoms in the 1,3,4-thiadiazole ring system), may facilitate the phenomenon of ESIPT.

More specifically, in the case of FABT molecules the dual fluorescence effect is also observed in non-polar solvents. Under such conditions, the dual emission is produced mainly by ESIPT processes, while aggregation-related phenomena are of lower importance, although present. They may enhance the effect, as demonstrated also in this paper where while the effect of dual fluorescence is already observed for low concentrations of FABT in a chloroform medium, the intensity of the longwave band can be significantly increased by aggregation effects.

Therefore, in order to understand our earlier deliberations pertaining to the phenomenon of dual fluorescence, in the present study we focused on the aforementioned compounds, specifically FABT, which was already discussed in our earlier papers 2015 [4], and a new 1,3,4-thiadiazole derivative where, in order to better illustrate the relevant fluorescence effects, we blocked the –OH group from the resorcylic ring (in the *ortho* position) to prevent this molecule from participating in excited state proton transfer. The core structures of both molecules, namely the resorcynyl and thiadiazole moieties, are identical.

## 2. Results

**Scheme 2:**
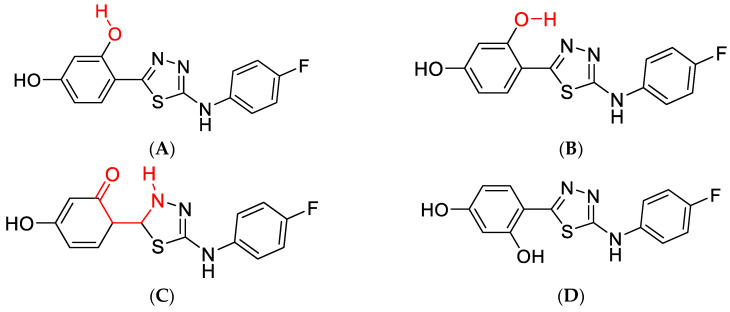
Keto-enol tautomerism and intramolecular hydrogen bonds in FABT: (**A**) enolic form which does not favor the formation of intramolecular bond with thiadiazole nitrogen atom; (**B**) enolic form favoring the formation of intramolecular bond with nearest thiadiazole nitrogen atom; (**C**) keto form; (**D**) enolic form favoring the formation of intramolecular bond with thiadiazole sulfur atom.

Scheme 1 presents the chemical structure of the compounds selected for this study: FABT and ADAPT. In its enol form, FABT contains a typical resorcylic system (Scheme 1A) and has the ability to create an intramolecular hydrogen bond between the hydroxyl group in the *ortho* position in the resorcylic ring and the nearest nitrogen atom in the 1,3,4-thiadiazole ring (Scheme 2). Meanwhile, ADAPT was synthesized in such a way that by employing the process of acetylation, we were able to block the formation of intramolecular hydrogen bonds between the aforementioned groups, while retaining the capacity for interactions with other molecules and solvent molecules by forming hydrogen bonds. As for the final element of the structure, the FABT molecule contains a fluorobenzene system and ADAPT an acetyl group, but these components of the molecules are mostly insignificant in the context of the subject matter discussed herein.

### 2.1. Analysis of Spectroscopic Effects

**Figure 1:**
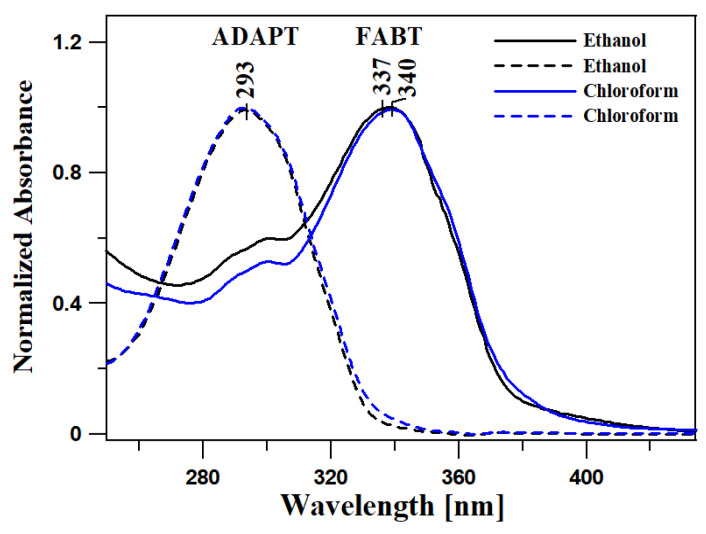
The electronic absorption spectra of the two compounds, FABT and ADAPT, registered in polar solvent—ethanol and non-polar solvent chloroform.

Figure 1 presents the electronic absorption spectra of the two compounds, FABT and ADAPT, registered in ethanol (EtOH—polar solvent) and chloroform (non-polar solvent). The wavelengths of absorption and fluorescence emission maxima, together with the refractive indices and dielectric constants ε recorded in various solvents, are presented in Tables S1 and S2 of the supplementary materials. The solvent polarity changes, result in a slight, but noticeable—by several nm, bathochromic shifts of the bands registered for the analyzed compounds, for FABT Δλ = 6 nm (522 cm^−1^) and for ADAPT Δλ = 6 nm (694 cm^−1^) (Figure 1 and Table S1 in Supplementary Materials (SM)). Noticeable shifts of the absorption band maximum indicate the analogues’ sensitivity to even the slight changes in medium polarity. Moreover, in all the FABT absorption spectra, we could observe a wide band associated with the S_0_→S_1_ transition within the range from 310 to 400 nm, with the maximum at approx. 330–350 nm, characteristic of the π→π* electronic transition. In the case of ADAPT, we observed a clear dominance of the band with the maximum at approx. 290 nm associated with the S_0_→S_1_ transition, within the range from 260 to 340 nm, in this case also characteristic of the π→π* electronic transition in the molecule, values of the molar extinction coefficient ε = 20000–25000 cm^−1^ M^−1^ and higher. The most notable observation at this stage, very significant in the context of the further analysis of the effect discussed, is the fact that the locations of the aforementioned absorption spectra changed only very slightly despite the relatively radical change in solvent polarity. This clearly evidences the relatively high stability of the ground state of any enol form (S_0_) depending on polarity changes of the surrounding medium.

**Figure 2:**
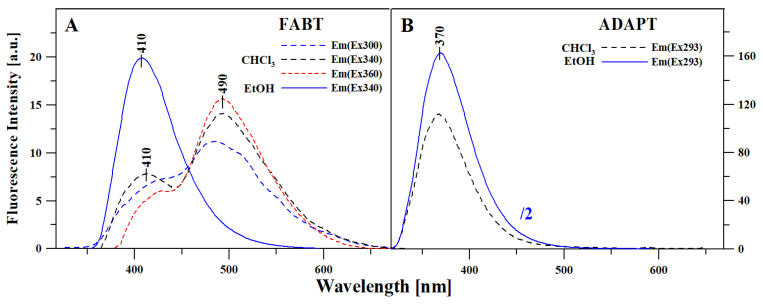
Fluorescence emission spectra for FABT (Panel (**A**)) and ADAPT (Panel (**B**)) in ethanol and chloroform. The excitation is pointed out in the figure for each sample.

Table S1 presents the measurements of the fluorescence emission spectra for the two compounds, FABT and ADAPT, corresponding to the absorption spectra from Figure 1. Compared to the ground state spectroscopic properties of the analyzed thiadiazoles, their excited state spectroscopic properties prove to be significantly more interesting. As can be seen in Panel A, with changing polarity of the solvent, FABT yielded either a single fluorescence emission band or a very specific and interesting effect of dual fluorescence, which is usually accompanied by the aggregation-related decrease in fluorescence intensity. Figure 2 in Panel A presents the fluorescence emission spectra for FABT in ethanol, where a single emission band was observed with the maximum at approx. 410 nm, and in chloroform, where even with different excitations, we observed the effect of dual fluorescence. The two emission bands for FABT, with the maxima at, respectively, ~410 and ~490 nm, were the most clearly visible with excitation at 340 nm, i.e., corresponding to the maximum of the main absorption band. The slight fluctuations in the fluorescence intensities in chloroform solution of FABT are most likely excitation wavelength-dependent, which is consistent with our former investigations into the aggregation effects. With increasing excitation within the band which, according to the theory of exciton splitting, corresponds to the possibility of various aggregation forms emerging, we observed increasing intensity of longwave emissions. However, in this case, the effect of dual fluorescence was already registered for very low concentrations and in a non-polar medium. Figure 2 in Panel B presents the fluorescence emission spectra for ADAPT in ethanol and chloroform, but in this case with even a significant change in medium polarity, we observed only the single fluorescence emission spectrum with the maximum at 370 nm. Moreover, the intensity of the emission band in the case of ADAPT in ethanol was divided by factor 2 due to the compound’s high quantum efficiency related to the binding of specific function groups to the molecule in the process of acetylation. It is therefore possible that in the case of ADAPT, the replacement of the –OH and –NH_2_ groups with acetyl substituents significantly limited the molecule’s capacity for interactions promoting non-radiant ways of dissipating the excitation energy (i.e., intermolecular hydrogen bonds), which simultaneously led to the increased quantum efficiency of fluorescence.

Based on our earlier deliberations pertaining to the phenomenon of dual fluorescence, we put forward a research hypothesis regarding the association between the dual fluorescence observed in the analyzed compounds and a number of contributing factors [2,4,5,17,20]. We noticed that for the phenomenon of dual fluorescence to emerge in the relevant analogues, a number of conditions have to be met. The first requirement is the correct conformation of the molecule, with the –OH group from the resorcylic ring—in the *ortho* position—located on the side of the nitrogen atom from the 1,3,4-thiadiazole ring—what we referred to as “N” conformation. The second key factor seemed to be associated with aggregation, i.e., given the correct conformation of the molecule, aggregation may lead, as also confirmed by Time-Dependent Density Functional Theory ([TD]DFT) calculations, to a specific intermolecular charge transfer. Subsequently, due to the possibility of the specific CT, adequate electronic states of CT character may emerge, and it is to the emission from those states that the long-wavelength fluorescence emission with the maximum at approx. 490 nm is related.

However, in the light of our new, previously unpublished results related to the emergence of dual fluorescence emission, as observed in non-polar solvents such as, e.g., chloroform (Figure 2A) or toluene (Figure S2 in SM), we can now positively associate the effect of dual fluorescence observed in this group of analogues with the process of excited state intramolecular proton transfer. Similar systems are often mentioned in literature reports where the cause of the dual fluorescence effect is identified as ESIPT [46,47,48,49,50,51]. Long-wavelength emission is often observed in molecules which have the ability to form an intramolecular hydrogen bond between the proton donor and acceptor (Scheme 2B). Such emission is most commonly related to the formation of a tautomer in the process of excited state intramolecular proton transfer (Scheme 2C). Moreover, it is noteworthy that with the increasing polarity of the solvent, we can clearly observe a hypsochromic shift of the longer fluorescence emission (Figure S2 in SM; this, however, is not observed in all solvents and the respective bands show slightly varying width). This fact clearly points to the complex nature of excited states which entails lower polarity than that of the corresponding ground states. This trait is characteristic of ketone tautomer emissions produced in excited states. Hence, as follows from descriptions in literature in the context of some molecules showing similar effects, the longer wavelength in the emission spectrum may be associated with the excited ketone tautomer, whereas the shorter wavelength originates from the emissions of the enol form which is better stabilized in polar environments due to the formation of hydrogen bonds with the molecules of the surrounding medium.

### 2.2. Quantum Mechanical Calculations—[TD]DFT

The abovementioned assumptions based on experimental data were further corroborated by quantum mechanical calculations, which clearly indicated the occurrence of the ESIPT process in FABT molecules. The energy discrepancy between the enol and keto form was 7.5 kcal/mol and was high enough to suggest that the only populated form of the system was its enol form (Figure S14). The absorption spectrum was dominated by the energy profile and intensity of enolic excited states. Table S7 juxtaposes the calculated vertical transfer energies and band positions in the experimental spectrum. If the electronic and oscillative structure of the transfers is governed by Franck–Condon principle, the vertical transfer energies should correspond to the centers of the experimental bands.

A key factor in explaining the effect of dual florescence is the similarity of the energy minima for the first excited state of the enol and keto forms (Figure S14). Consequently, the proposed model of the phenomenon is as follows: first, the enol form is excited; this is followed by relaxation to the lowest vibrational level of the enol form excited state or the alternative process of hydrogen transfer of the –OH group to the nitrogen atom, with the accompanying formation of the keto form and its subsequent relaxation to the lowest oscillative level of the excited state. This is followed by fluorescence from the minimum corresponding to either the enol form (higher energy) or keto form (lower energy), and the resulting return to the ground state. Eventually, what follows is non-radiant relaxation of the keto form to ground state enol. The energy of the transition state (TS) between the excited enol and ketone forms is such that for the given excitation wavelength, the transfer takes place virtually without a barrier.

The energy (in kcal/mol) of the respective states is presented in the SM, Table S9.

Assuming Franck–Condon proximity applies, the vertical fluorescence energies should correspond to the centers of the respective bands in the experimental spectrum. As is clearly visible, the calculations predict the emergence of a double maximum while slightly (although acceptably) underestimating the split in fluorescence bands.

It is also noteworthy that if the proposed mechanism of excited state tautomerism should correspond to the physics of the problem, one should expect the low-energy fluorescence band to be quenched in both base (detachment of the labile proton by –OH ions) and acidic medium (blocking of the nitrogen atom, as observed in this study).

### 2.3. Excitation Spectra

**Figure 3:**
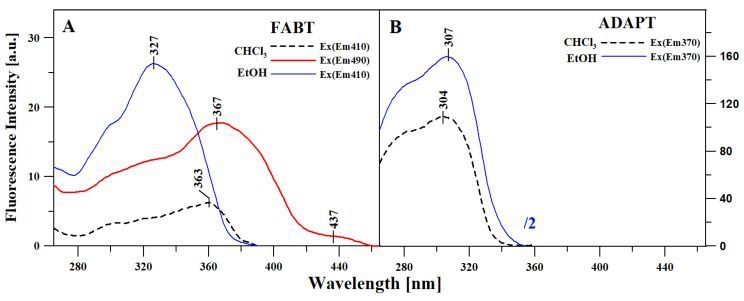
Fluorescence excitation spectra for FABT (Panel (**A**)) and ADAPT (Panel (**B**)) in the same selection of solvents as in Figure 1 and Figure 2. For FABT (Panel A), emission was registered for the wavelengths of, respectively: 410 nm for ethanol and at 410 and 490 for chloroform. In the case of ADAPT (Panel B), emission was registered for the wavelength of 370 nm in both solvents.

Figure 3 presents results corresponding to those presented in Figure 1 and Figure 2, i.e., the fluorescence excitation spectra (Ex) for FABT (Panel A) and ADAPT (Panel B), in the same selection of solvents as above. For FABT (Panel A), emission in the event of excitation was registered for the wavelengths of, respectively: 410 nm (the maximum of the first fluorescence band from the emission spectra Em) for ethanol, and at 410 and 490 for chloroform (excitation at 490 nm corresponded with the long-wavelength maximum from the fluorescence emission spectra). In the case of ADAPT (Panel B), emission in the event of excitation was registered for the wavelength of 370 nm in both solvents. Fluorescence excitation spectra, due to their high selectiveness, facilitate in-depth characterization of specific energy levels, which in turn produce specific types of emissions. So, for ADAPT (Panel B) we did not observe other changes, apart from the noticeable change in fluorescence intensity. Whereas in the case of FABT (Panel A), the changes observed were considerably more significant. In terms of the FABT fluorescence excitation spectra, there was a band with the maximum at 327 nm (in ethanol) and a band shifted significantly towards the long-wavelength side with the maximum at ~363 nm (with shortwave excitation) and with the maximum at 367 nm (with long-wavelength excitation) in chloroform.

Based on our previous studies, in which we used only polar media, it is clear that these effects most closely corresponded to the various aggregated forms of FABT and other thiadiazole molecules, which was also observed in the case of resonance light scattering (RLS) spectra (Figure S3). However, even in our earlier studies we clearly pointed out that aggregation effects could not fully explain the effects related to the dual nature of fluorescence revealed in the compounds. Moreover, in this case the effects related to dual fluorescence are observed already for very low concentrations of the compounds and in polar solvents. This conclusion was further confirmed by the observed RLS spectra, showing high intensity even in the case of solvents where dual fluorescence did not emerge. Changes in terms of excitation spectra (at differing excitation wavelengths) can now be, in accordance with standard literature determinations, clearly associated with the *cis*- and *trans*-enol molecular forms, designated in our previous publications respectively as the “N” conformation and “S” conformation. The shift towards the longer wavelengths observed in the *cis*- form compared to the *trans*- form is due to the existence of an intramolecular hydrogen bond. Our previous studies conducted on crystalline systems confirmed that the *cis*- form is more stable than *trans*-, as it allows for the formation of intramolecular hydrogen bonds. Furthermore, the fact that emissions from excited keto forms have a longer wavelength compared to emissions from the enol form signifies that the energetic differences between the excited and ground states are lower in the case of keto, as compared to enol forms. This was confirmed by calculations conducted using two distinct methods, which allowed us to estimate the dipole moment differences between the respective excited and ground states.

Analogically to the emission spectra, the absorption spectra of the ketone tautomer are also shifted towards longer wavelengths as compared to the absorption spectra of enol forms. Hence, as already reported in our earlier papers, the absorption spectrum with the maximum at 340 nm corresponds to the enol form (the one with the predominance of *cis*- conformation in previous reports—the “S” conformation). It is particularly worth emphasizing that the notable differences between the absorption and fluorescence excitation spectra point to the significant structural differences between the absorbing and emitting species. These observations are in line with our previous crystallographic studies where the monocrystals structures obtained varied depending of the solvent used. In more detail, depending on the solvent, various rotational conformers of the resorcylic ring relative to the thiadiazole ring heteroatoms (S and N) were formed. Moreover, a notable long-wavelength “tailing” of the bands present in the FABT excitation spectra gives further evidence for the involvement of aggregation-related processes together with further evidence given by RLS measurements (Figure S3). However, it should also be clearly emphasized that the processes of aggregation only serve to enhance the effect which can also emerge without aggregative contribution.

Figure S3 (in SM) presents a comparison between resonance light scattering (RLS) spectra for FABT (Panel A) and ADAPT (Panel B) in the same solvents as in the previous figures. It can be seen that in the case of FABT in chloroform we observed a significantly higher intensity of the RLS spectra when compared to FABT in ethanol. However, that is not to say that RLS spectra were not present in ethanol at all, even though their intensity was significantly reduced. Moreover, Panel B indicates that RLS bands were present even for ADAPT in the selected solvents, but with a significantly lower intensity. In our earlier studies focused on the RLS spectra in different solvents where the discussed fluorescence effects were observed, RLS spectra were present regardless of whether the effect occurred or not, and with a significantly greater intensity for that matter. Therefore, it is clear that aggregation is a factor that emerges in this type of molecules, both in cases where single and double fluorescence emission is observed. Consequently, as we have already argued in our previous papers on the topic, aggregation by itself cannot be a sufficient explanation of the dual fluorescence phenomenon which seems to be also related to the ESIPT effect. Therefore, the RLS spectra give evidence that in some cases and most likely at higher concentrations, the ESIPT and AIE processes may superimpose and both may contribute to the dual fluorescence effects observed. In other words, in low concentrations, the process of ESIPT itself is capable of generating dual fluorescence, while aggregation processes in adequate media can significantly enhance the same, as confirmed in our earlier studies on the subject.

Moreover, the measurements in various solvents revealed notably lower fluorescence quantum yields in FABT compared to those in ADAPT (see Table S6), which is evidence for the involvement of aggregation in the dual fluorescence effects observed. In more detail occurs and particularly in FABT molecule, the lower the fluorescence quantum yield, the easier the dual fluorescence. Let us notice that the lower quantum efficiency confirms the possibility of a relatively high contribution of aggregation to the observed dual fluorescence effect. Dual emission in FABT is observed only in cases where quantum efficiency is very low. This clearly points to aggregation as the main factor enhancing the ESIPT-induced dual emission, which will also be discussed further in the paper in the context of concentration effects.

**Figure 4:**
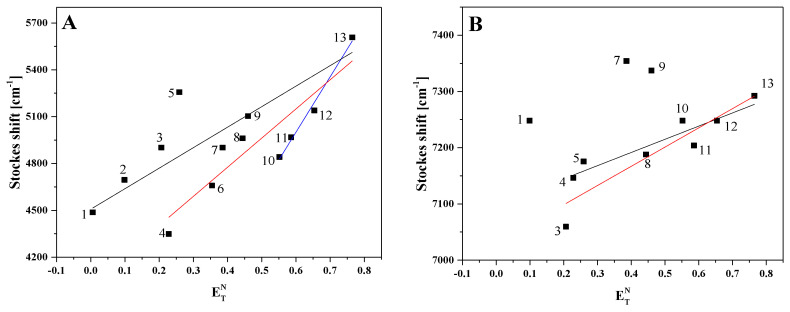
Panel (**A**): Stokes shift variation with normalized value of solvent polarity for FABT. Panel (**B**): Stokes shift variation with normalized value of solvent polarity for ADAPT dissolved in different solvents (1—cyclohexane, 2—toluene, 3—THF, 4—ethyl acetate, 5—chloroform, 6—acetone, 7—DMF, 8—DMSO, 9—acetonitrile, 10—propan-2-ol, 11—butan-1-ol, 12—ethanol 13—methanol).

At the next stage of the study on the ESIPT effects observed in the two molecules tested in our work, with a particular focus on FABT, we estimated the changes of the ground and excited state dipole moments for the relevant molecules. The estimation relied on a method based on the solvatochromic phenomenon. The change in ground and the excited dipole moments were estimated through use of the microscopic solvent function $E_{T}^{N}$ Equation (3)) for different solvents such as methanol, ethanol, butan-1-ol, propan-2-ol, acetonitrile, ethyl acetate, DMSO, DMF, THF, acetone, toluene, cyclohexane and chloroform. The graphs of Stokes shift from the microscopic solvent function $E_{T}^{N}$ parameter are presented in Figure 4, panels A and B. The slopes of the fitted lines, Onsager cavity radius, coefficient of determination and dipole moments parameters for the studied thiadiazoles are given in Table 1.

In this method based on the Dimroth–Reichardt methodology, the changes in dipole moment Δµ in FABT were estimated for three linear fits and were, respectively, 2.098 D (r^2^ = 0.79) and 1.760 D (r^2^ = 0.92) for mixed solvents and 2.893 D (r^2^ = 0.98) for polar protic solvents, whereas in the case of ADAPT, the values of 0.891 D (r^2^ = 0.86) and 1.157 (r^2^ = 0.87) were obtained for mixed solvents. In the analyzed compounds, the excited state dipole moment was higher than the ground state dipole moment, which corroborates the conclusion that the studied thiadiazoles are more polar in their excited singlet state compared to their ground state. In the molecular solvent polarity parameter intermolecular solute–solvent hydrogen bond donor–acceptor interaction, along with solvent polarity, has been included.

The results obtained using this method show that in case of the excited keto tautomer and its ESPIT-related effects, the difference between the ground and excited state will be much smaller compared to that characteristic of enol form of the compound. One should also note the relatively complex character of the correlations shown in Table 1 which clearly confirm the fact that depending on the character of the solvent, hydrogen bonds may form which, given a suitable medium, facilitate aggregation effects.

In the subsequent stage of our study on the effects of dual fluorescence emission in the selected 1,3,4-thiadiazole analogues, time-resolved measurements of the fluorescence lifetime were conducted. The results were recorded for excitation with 294 nm wavelength with the use of a F360 nm emission filter. Example lifetime decay curves were presented in Figures S10 and S11, while Table 2 and Table 3 present the detailed measurement results. For ADAPT, we observed a single decay in both chloroform and ethanol with similar lifetime values (Figure S11 and Table 3). For FABT, a double decay result was obtained in ethanol, with the lifetime of approx. 1.59 ns and 0.79 ns (Figure S10 and Table 3), whereas in chloroform, the best fit was obtained for three lifetime constituents, with one corresponding to that observed for FABT in ethanol (1.67 nm) and another of approx. 0.22 ns. The corresponding ethanol and chloroform lifetime values (1.79 and 1.67 ns) are the lifetimes of normal fluorescence (with a single exponential decay) originating from the enol form, while the very short component (clearly visible in the decay—Figure S10—of approx. 0.22 ns) corresponds to the *keto* tautomer. Therefore, the obtained fluorescence lifetimes clearly corroborate the attribution of shorter wavelengths (in the fluorescence emission spectra) to the *enol* tautomer, and longer wavelengths (in the fluorescence emission spectra)—to emissions originating from the excited ketone tautomer of the studied molecule. The short fluorescence lifetime component characteristic of the excited keto-tautomer originates from the fact that in polar solvents the formation of such tautomer may occur relatively easier compared to that in polar solvents, where the various intermolecular interactions between the compound and solvent make the formation of keto-tautomer less likely.

It is worth emphasizing that the analysis of fluorescence lifetime for FABT in chloroform also identified a third, very long constituent of approx. 27.3 ns, which may be due to factors of which one is the possibility of aggregation of interacting molecules. In this particular solvent, the intermolecular interactions between solvent and molecules of the investigated compound are less favored compared to those occurring between the molecules of the compound itself. Similar effects were evidenced to occur even in highly polar environments, including water, and were reported in our previous studies [52].

**Figure 5:**
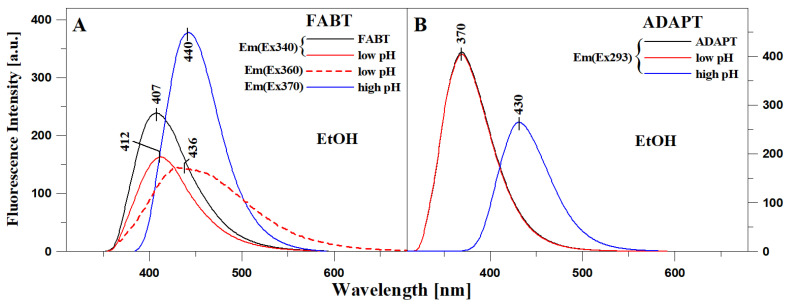
Emission fluorescence spectra corresponding to the absorption spectra in Figure S7 for FABT (Panel (**A**)) and ADAPT (Panel (**B**)).

In the final stage of the study, we analyzed the impact of very significant changes in the medium pH on the fluorescence effects in selected 1,3,4-thiadiazole analogues. In this series of experiments, we aimed at determining whether forcing the emergence of specific ionic forms (such as anionic or cationic, whose was conformed in our earlier crystallographic research) would trigger the prevalence of aggregation effects and force the occurrence of dual fluorescence, or whether we would only observe single shifted emission bands. To that end, Figure S7 presents the electronic absorption spectra for FABT (Panel A) and ADAPT (Panel B) in ethanol, as well as in ethanol after acidification to pH~1 (so-called apparent pH) and at pH~10 after the addition of base. It could be observed that in the case of ADAPT (Panel B), the spectra in ethanol and ethanol with the addition of acid are practically the same (apart from differences in intensity), with a clear maximum at ~293 nm; we also observed no band widening that could evidence aggregation effects. The spectrum obtained after the addition of base was clearly shifted towards the long-wavelength side with the maximum at approx. 343 nm. Similar effects were observed for FABT (in Panel A), in this case the spectrum obtained after the addition of acid showed reduced intensity and the band was clearly widened at approx. 360 nm, which signifies the presence of aggregation effects. After the addition of base and deprotonation of the –OH group (in the *ortho* position in the resorcylic ring), the band was bathochromically shifted to approx. 370 nm. As can be observed further in Figure 5 presenting the emission fluorescence spectra (with excitation at the maximum of the absorption spectrum) corresponding to the absorption spectra in Figure S7, radical pH modifications triggered corresponding changes in the emission spectra. In the case of ADAPT, in both ethanol and ethanol acidified to low pH levels, the spectrum remained virtually unchanged. However, after the addition of base, it was shifted to ~430 nm and its intensity was significantly lowered. On the other hand, in FABT, the spectrum obtained in ethanol and ethanol acidified to low pH (with excitation within the range of the absorption spectrum widened on the long-wavelength side, ~360 nm) was significantly less intensive and shifted towards the long-wavelength side to ~436 nm. The result confirms the existence of aggregation effects in the analyzed systems and simultaneously signifies that they are not solely responsible for the aforementioned effects related to the molecules’ dual nature. This follows from the results of our previous studies which revealed that at low pH, we could observe binding to the N atom (in the 3rd position in the 1,3,4-thiadiazole ring) of the ionic form characteristic of low pH (our earlier studies were crystallographic). Notably, the band with the maximum at 436 nm may originate from this particular ionized form of the FABT molecule—a fact that was not discussed in our earlier publications. Meanwhile, with the phenomenon of deprotonation of the –OH group from the resorcylic ring, the emission band was shifted towards the long-wavelength side with the maximum at ~440 nm. The band was therefore shifted towards longer wavelengths relative to the emission spectrum of the enol form, and at the same time towards shorter wavelengths relative to the emission spectrum of the excited ketone tautomer—present in non-polar solvents. Hence, due to deprotonation of the –OH group, the molecule loses its capacity to form an intramolecular hydrogen bond, which means that ESIPT-related effects cannot occur and neither can the phenomenon of dual fluorescence emission. So, the analysis of a single band originating from ionic forms also clearly corroborates the association of effects related to dual emission with the ESIPT process. In the case of ionic forms with the emerging –N–H^+^ group, the possibility of direct charge transfer in the molecule was also blocked and the effects of dual fluorescence were not observed; however, the band was significantly shifted towards the long-wavelength side.

The occurrence of the respective anionic and cationic forms is confirmed even more clearly by pH-metric titration experiments (Figures S15 and S16). Figure S15 shows electronic absorption spectra of FABT and ADAPT recorded at pH range of ~1 to 12. Additionally, the respective titration curves together with marked pH points, relevant to the ionization of of –OH and N–H into the respective –O^−^ and N–H^+^. Figure S16 shows the fluorescence emission spectra of FABT and ADAPT related to the absorption spectra given in Figure S15. Clearly, the ionization of –OH group affects the long wavelength shift of the absorption maxima. The increase in acidity is accompanied with the protonation of thiadiazole nitrogen and the formation of -NH^+^, which manifests in hypsochromic shift of the absorption maxima. Similar effects were observed previously (as illustrated in Figure S7). It is also worth mentioning that in aqueous solutions, the absorption maxima changes are affected by much stronger molecular aggregation processes and the aggregation-related effects are even more pronounced in the fluorescence emission spectra. Figure S16 clearly shows that the change in pH of the aqueous solution is accompanied with the appearance of ESIPT-related bands (long wavelength emission) or the band characteristic of the ionized (deprotonated) –OH group (~430 nm). As noticed, in the case of fluorescence emission spectra recorded in aqueous media, strong aggregation effects are observed, which at certain pH values strengthens the processes observed, and manifests as a decrease in intensities of the emission bands (the effect is much stronger compared to that given in Figure 5). Therefore, the results depicted in Figures S15 and S16 are consistent with those presented in Figure 5 and Figure S7.

**Figure 6:**
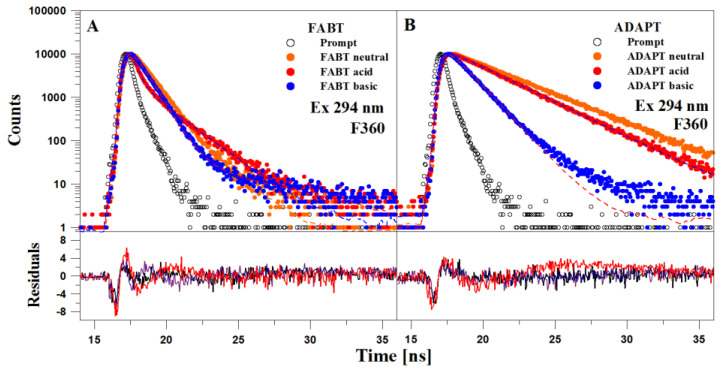
Panel (**A**). TCSPC analysis for FABT in EtOH and EtOH with the addition of acid and base (apparent pH). The top panel presents the decay of fluorescence intensity. Black color denotes the apparatus profile. Points correspond to measurement results and the continuous line to the double component analysis. The bottom panel presents the distribution of residuals. The excitation wavelength was 294 nm. Panel (**B**). TCSPC analysis for ADAPT in EtOH and EtOH with the addition of acid and base (apparent pH). The top panel presents the decay of fluorescence intensity. Black color denotes the apparatus profile. Points correspond to measurement results and the continuous line to the double component analysis. The bottom panel presents the distribution of residuals. The excitation wavelength was 294 nm, fluorescence emission was recorded for wavelength >360 nm (emission filter F360).

Subsequently, time-resolved fluorescence lifetime measurements were performed in an experiment analogous to that discussed in Figures S10 and S11. Figure 6 (Panel A—FABT and Panel B—ADAPT) presents the values of lifetime decay for excitation at 249 nm with an F360 filter in both molecules, whereas Table 2 and Table 3 present detailed lifetime values obtained in the experiment. As can be observed, both in the case of FABT and ADAPT, the fluorescence lifetime vales varied significantly for the relevant ionic groups (anionic form and form ionized with the –N–H^+^ group). In ADAPT, for a given form we obtained a single fluorescence lifetime, whereas in FABT, for virtually every ionic and non-ionic form we obtained two different lifetimes; fit with only a single component was not possible. This proves that there is a relative fluidity of equilibrium between both forms. At the same time, the effects confirm that the dual fluorescence emission observed in non-polar solvents as well as—in previous studies—in aqueous media, definitely originates from the excited ketone tautomer. In the case of FABT, e.g., for the neural form in ethanol, the lifetime is ~1.585 ns (and the shorter one—0.794 ns); in acidified ethanol the values were, respectively, 4.994 ns (and 0.73 ns), whereas for the anion form it was 1.79 ns (and 0.22 ns). The noticeable extension of the lifetime of the anionic form with the –N–H^+^ group (to as much as 5 ns) clearly suggests increased energy demand in the process of the form’s emergence in the ground state. It is clear that depending on the pH the two ionic forms may occur, namely the –O^−^ or –N–H^+^.

The measurements of fluorescence excitation spectra (Figure S8 in SM) corresponding to the results presented above (Figure S7, Figure 5 and Figure 6) corroborate our hypotheses pertaining to the existence in the particular pH range of specific ionic or non-ionic forms for which, rather than dual fluorescence, we can only observe significant shifted emission bands associated with the given ionic form of the molecule, which do not overlap with the range of maxima (long-wavelength) established for the FABT molecule. On the other hand, the RLS spectra presented in SM in Figure S9 (corresponding to the Figure S7, Figure 5 and Figure 6) corroborate the possible existence of aggregated forms of the molecules even in media where the effects related to dual fluorescence emission are not observed. The emergence of said ionic forms had already been confirmed in our studies conducted on crystalline systems, but in order to confirm their presence in various solvent environments with the addition of acid or base, Figures S4 and S6 present the FTIR spectra obtained from an experiment analogous to that presented in Figure S7, Figure 5 and Figure 6 (Tables S3 and S4). We could clearly observe changes in the infrared spectra for FABT in ethanol relative to those recorded for FABT in ethanol with the addition of acid or base (Figure S4), particularly in terms of the bands characteristic of C=N stretching vibrations (from the 1,3,4-thiadiazole ring). We noted a clear decrease in their intensity as the proton was bonded to the nitrogen atom in the 3rd position from the resorcylic ring. Meanwhile, during the formation of the anionic FABT form in the base medium, the changes were more evident, an –O^−^ group was formed in the resorcylic ring, which shifted the band from 1622 to 1641 cm^−1^, the hydrogen bond in the molecule disappeared and the vibration in this range became significantly more intensive. Moreover, a significant increase in intensity of bands in base treated FABT sample (1554, 1433, 1305 and 1072 cm^−1^) clearly points to the increased tendency for aggregation. Moreover, an increased tendency for aggregation is accompanied with an increase in hydrophobicity of the environment. This shifts the equilibrium from *trans*-enol to *cis*-enol, which results in an increased emission from the enol tautomer.

For ADAPT (Figure S6), the changes, particularly with regard to ADAPT in ethanol and ADAPT in acidified ethanol, were less pronounced as in this case, the respective groups were blocked in the process of the compound’s synthesis. Additionally, Figure S5 presents the Raman spectra for FABT in ethanol, and Table S5 contains the respective vibrations associated with specific functional groups. As can be seen, the Raman spectra fully confirmed the presence in the enol form of bands with the maxima at 1634 and 1614 cm^−1^ associated with stretching vibrations of the C=N group.

To recapitulate the results of the above measurements, it would be prudent to refer to our earlier results in cases where the emission spectra showed the effect of dual fluorescence emission with growing prevalence of aggregation factors in a given medium. As follows from the results of the above experiment, aggregation changes the character of interactions in the solvent by involving larger numbers of molecules in the interaction processes, whereby the strength of intramolecular hydrogen bonds yields to aggregational interactions with both other molecules of the compound itself and molecules of the solvent. However, in the above case, by correctly and fairly intuitively adjusting the medium properties, we were able to demonstrate that by itself, aggregation is insufficient to fully account for the observed effects. It is worth emphasizing that the ESIPT process observed in non-polar solvents is the main factor responsible for the dual emission in FABT and other structurally similar compounds. Nevertheless, the aggregation effects may also occur in non-polar solvents and may contribute to the emergence of dual emission. In polar solvents, the dual fluorescence emission may be induced by the aggregation, which in such conditions seems mandatory for the said dual fluorescence effects to occur. This hypothesis is supported by our previous findings from investigations into the behavior of the thiadiazole derivatives in polar solvents with varied “apparent pH” [1,2,3,4,5]. Protonation of the molecules due to changes in the environment resulted in the emergence of an additional band by blocking the possibility of a new hydrogen bond forming in the analyzed molecule. The emergence of forms with the –N–H^+^ group also led to the corresponding shift of the emission band and significant extension of the lifetime. Meanwhile, using a non-polar solvent—and hence limiting interactions with solvent molecules—facilitated the processes related to excited state intramolecular proton transfer. In turn, this process was responsible for long-wavelength emission in selected 1,3,4-thiadiazole analogues, and in FABT in the present experiment.

To recapitulate the discussion on the studied effects, one should also emphasize the very significant contribution of aggregation effects, which may also induce the effects related to dual fluorescence emissions (AIE fluorescence), or more specifically enhance the relevant ESIPT-triggered effects. Let us note the data presented in Figures S12 and S13, which illustrate the electronic absorption spectra in FABT and ADAPT (Figure S12), as well as the corresponding fluorescence emission spectra (Figure S13) in butan-1-ol and chloroform, relative to the changing concentration of the compound. As can be observed for both FABT and ADAPT, increasing the concentration triggers no changes in the absorption spectra other than the increase in absorbance. Correspondingly, in the case of emission spectra in butanol, only increased emission intensity is apparent. However, very interesting effects can be observed for the emission spectra in chloroform, particularly in the case of FABT. For ADAPT, not unlike in butanol, only an increase in band intensity was recorded. But for FABT in chloroform, we observed a larger increase in the intensity of the band related to longwave fluorescence emission. This is clearly visible in Figure S13, Panel C, which presents the ratio of the second to the first emission with increasing compound concentration. Polarization and the protonic character of the solvent facilitate *trans*-enol transformation, as discussed above. However, in aggregation processes, molecules are not fully surrounded by the solvent and therefore have limited exposure to the molecules thereof. Consequently, changes in the medium hydrophobicity are possible, which results in the enhancement of ketone tautomer emissions. As a result of aggregation of *cis*-enol molecules which include an intramolecular hydrogen bond, longwave emission is intensified. Hence, aggregation aided by the correct change in molecular conformation enhances the ESIPT process and therefore the effects related to dual fluorescence emission. Those effects will be further carefully analyzed in our subsequent works related to this topic. The effect of dual fluorescence occurring in selected analogues from this group of compounds can therefore be observed even in very low concentrations in non-polar solvents. The interpretation of fluorescence excitation spectra, fluorescence lifetimes and quantum efficiency values clearly and patently points to the ESIPT process as the source of said effects. At the same time, aggregation processes occurring in adequate media may serve to strongly enhance the effect, as confirmed by literature data as well as the result presented herein.

## 3. Materials and Methods

### 3.1. Materials

Synthesis of ADAPT. 2,4-dihydroxybenzoic acid (2.50 g, 16.00 mmol) was reacted with thiosemicarbazide (1.48 g, 16.00 mmol) in the POCl_3_ medium (10 mL). The mixture was stirred overnight at 75 °C, then cooled down to 40 °C, and the excess POCl_3_ was carefully quenched by the addition of water. The mixture was then refluxed for 5 h and cooled down to ambient temperature. The pH was adjusted to slightly basic using a saturated NaOH solution. The obtained solid was filtered off, rinsed with water and allowed to dry in air. Dry powder was suspended in acetic anhydride (20 mL) and a few drops of concentrated H_2_SO_4_ were added. The mixture was then refluxed for 6 h. The product was filtered off, rinsed with water and recrystallized from ethanol to produce ADAPT in the form of an off-white powder with 83% yield. The structure of the compound obtained was then confirmed with NMR spectroscopy (Figure S1). Synthesis of FABT. This compound was synthesized according to the previously published procedure [53].

Stock solutions of the two thiadiazols FABT and ADAPT were prepared by dissolving approximately 1 mg of the respective compounds in selected solvents (methanol, ethanol, butan-1-ol, propan-2-ol, acetonitrile, ethyl acetate, DMSO, DMF, THF, acetone, toluene, cyclohexane and chloroform). All the solvents used in this study were of analytical grade. An appropriate volume of the solution was added to 2 mL of solvent in order to obtain the required absorbance intensity. The molar concentrations of FABT and ADAPT dissolved in EtOH and DMSO were as follows: C_1_ = 6.6 × 10^−5^ M, C_2_ = 3.3 10^−5^ M, C_3_ = 7,8 × 10^−5^ M and C_4_ = 5.2 × 10^−5^ M.

### 3.2. Methods

#### 3.2.1. Electronic Absorption and Fluorescence Spectra

The UV-Vis spectra were recorded using a Cary 300 Bio (Varian, Santa Clara, CA, USA) spectrophotometer with a tray holder thermostatted by 6 × 6 multi-cell Peltier block. A thermocouple probe (Cary Series II from Varian) placed directly in the sample was used for temperature control.

A Cary Eclipse spectrofluorometer (Varian) was used for fluorescence excitation, emission and synchronous spectra measurements at 22 °C. Fluorescence spectra were recorded at the resolution of 0.5 nm and corrected for the spectral characteristics of the lamp and photomultiplier. Resonance light scattering (RLS) measurements were recorded according to the previously reported protocol [54]. The excitation and emission monochromators of the spectrofluorometer were scanned synchronously (0.0 nm interval between the excitation and emission wavelengths); the slits were set to obtain a spectral resolution of 1.5 nm. Grams/AI 8.0 software (Thermo Electron Corporation, Waltham, MA, USA) was applied for the analysis of spectroscopic data.

#### 3.2.2. Time-Correlated Single Photon Counting (TCSPC)

FluoroCube fluorimeter (Horiba, Montpellier, France) was applied for time-correlated single photon counting (TCSPC) measurements. The pulsed Nano-LED diode at 294 nm (pulse duration of 700 ps) operated with 1 MHz repetition was used for sample excitation. In order to avoid pulse pile-up, the pulse power was adjusted using a neutral gradient filter. Fluorescence emission was recorded using a TBX-04 picosecond detector (IBH, JobinYvon, Glasgow, Scotland, UK). Data acquisition and signal analysis were carried out using the Data Station and DAS6 software (version 2.4), JobinYvon (IBH, UK). All fluorescence decays were measured in a 10 × 10 mm quartz tray using a 360 nm emitter cut-off filter. The excitation profiles required for the simplified analysis were measured without the emitter filters on a light scattering tray. All measurements were performed at 20 °C in various solvents. Each case of fluorescence decay was analyzed with the multiexponential model shown in the equation:

(1) It=∑iαiexp(−tτi)

where α_i_ and τ_i_ are the pre-exponential factor and the decay time of component i, respectively.

The best-fitted parameters were obtained by minimizing the reduced χ^2^ value as well as through residual distribution of the experimental data. The fractional contribution (fi) of each decay time was calculated with the following equation:

(2) $$f_{i}=\frac{\alpha_{i}\tau_{i}}{\sum_{i}\alpha_{j}\tau_{j}}$$

#### 3.2.3. Methodology and Calculation of Dipole Moments

The dipole moment of a molecule in the excited state can be determined using the solvatochromic method [55,56,57,58], which is dependent on the internal electric field effect. There are linear correlations between the location of absorbance and fluorescence maxima, with the solvent polarity parameters including the dielectric constant (e) and refractive effect (n) of the solvent. In this paper the change of dipole moments was calculated based on microscopic solvent parameters ($E_{T}^{N}$).

The effect of polarization and hydrogen bonding with solvents can be expressed by the function E_T_(30) which is useful for measuring solvent polarity by solvatochromic method with betaine dye as a probe solute [59]. The empirical solvent polarity parameter $E_{T}^{N}$ which was proposed by Reichardt correlates better with spectral shift than the traditionally used bulk solvent polarity functions (ε, n). The change in dipole moment can be expressed by the following equation (Equation (3)):

(3) $$\nu_{a}{-\nu }_{a}=m\cdot E_{T}^{N}+\mathrm{const}$$

where microscopic solvent polarity ($E_{T}^{N}$) is a normalized value of E_T_(30) and is defined using water ($E_{T}^{N}$ = 1) and tetramethylsilane ($E_{T}^{N}$= 0) as the extreme reference solvent with the equation (Equation (4)):

(4) $$E_{T}^{N}=\frac{E_{T}\left(\mathrm{solvent}\right){-E}_{T}\left(\mathrm{TMS}\right)}{E_{T}\left(\mathrm{water}\right){-E}_{T}\left(\mathrm{TMS}\right)}=\frac{E_{T}\left(\mathrm{solvent}\right)-30.7}{32.4}$$

The change in dipole moment can be determined as (Equation (5)):

(5) $${\Delta \mu =\mu }_{e}{-\mu }_{e}=\sqrt{\frac{81\;m}{{(6.2{/a}_{0})}^{3}11307.6}}$$

where m is the slope of the linear plot of $E_{T}^{N}\;$versus Stokes shift. The values of E_T_(30) and $E_{T}^{N}$ for the solvent are presented in Table S2.

#### 3.2.4. FTIR Measurements

Measurements of ATR-FTIR background-corrected spectra were carried out on the solvents using a HATR Ge through a (45° cut, yielding 10 internal reflections) crystal plate for liquids, and were recorded with a 670-IR spectrometer (Varian). Typically, 25 scans were collected, Fourier-transformed and averaged for each measurement. Absorption spectra at the resolution of one data point per 1 cm^−1^ were obtained in the region between 4000 and 400 cm^−1^. The instrument was continuously purged with argon for 40 min before and during measurements. The Ge crystal was cleaned with ultrapure organic solvents from Sigma-Aldrich Co (Saint Louis, MI, USA). All experiments were carried out at the temperature of 22 °C.

#### 3.2.5. Raman Measurements

Raman scattering spectra of the liquid samples placed onto a quartz plate (identical conditions as in the FTIR experiments) were recorded with the Via Reflex Raman Microscope from Renishaw (Wotton-under-Edge, UK), equipped with two holographic ultrahigh precision diffraction grating stages and a high sensitivity ultralow noise CCD detector. A 514.5 nm Ar+ ion laser was used to record Raman scattering. The spectra were accumulated within a 10 s integration time.

#### 3.2.6. Fluorescence Quantum Yield

The quantum yields of fluorescence of FABT and ADAPT solutions were determined using 7-diethylamino-4-methylcoumarin (coumarin1) as the fluorescence standard. The measurements were carried out in ethanol $\phi_{F}$= 0.73 [60,61,62]. The final fluorescence quantum yield values were calculated based on Formula (6),

(6) $$\phi_{F\left(X\right)}=\phi_{R\left(\mathrm{EtOH}\right)}\left(\frac{\lambda_{\mathrm{exR}\left(\mathrm{EtOH}\right)}}{\lambda_{\mathrm{exX}}}\right)\left(\frac{I_{X}}{I_{R\left(\mathrm{EtOH}\right)}}\right)\left(\frac{\eta_{X}^{2}}{\eta_{R\left(\mathrm{EtOH}\right)}^{2}}\right)$$

where the subscript X denotes corresponding FABT or ADAPT in different solvents, λ_ex_ is the value of absorbance at the excitation wavelength, I—the plane under the emission curve, and η the refractive index of the solvent. R—coumarin1, X—compound.

#### 3.2.7. Computational Details

All the computations were performed with the Gaussian 09 software package [63]. The presented results were obtained from DFT calculations [64] performed using one of the most popular exchange-correlation functionals—B3LYP [65]. The calculations took advantage of a good-quality functional base aug-cc-pVDZ [66], the numerical integration included in the computation procedure was conducted with the use of very fine grids (UltraFineGrid version 09 as termed in the Gaussian package). The solvent was described in the PCM model [67]. Excited states were described in accordance with the TDDFT formalism (Time Dependent DFT) [68]. The results were verified with the use of a higher-quality base—cc-pVTZ [66], the CAM-B3LYP functional [69], which facilitates the correct description of potential charge-transfer (CT) states as well as a non-standard variant of the TDDFT method—the so-called TDA-TDDFT (Tamm–Dancoff approximation).

#### 3.2.8. pH Measurement

Two thiadiazoles, FABT and ADAPT, were dissolved in 2 mL of distilled water, and then appropriate aliquots of this solution were added to 50 mL of distilled water. In order to obtain pH 12, 0.1 M of NaOH was added to each sample. Next, 1 M of HCl acid was gradually added resulting in a desirable pH of aqueous FABT and ADAPT solutions. The respective titration curve plots are given in Figure S15. All measurements were carried out at room temperature using the Elmetron CP-502 pH-meter.

## 4. Conclusions

The research results presented in this article, obtained with the use of stationary and time-resolved fluorescence spectroscopy, indicate the presence of dual fluorescence in the emission spectra of FABT in non-polar solvent media. In the case of the second analogue used in the study (ADAPT), the effect of dual fluorescence was not observed due to the fact that the possibility of forming a hydrogen bond between the –OH group and the nitrogen atom in the thiadiazole ring was blocked. As follows from the presented results of fluorescence, UV-Vis, infrared and Raman measurements, the phenomenon is closely related to the process of excited state intramolecular proton transfer and the resulting formation of a suitable ketone tautomer in the excited state. The preliminary quantum-mechanical [TD]DFT calculations corroborate the trend observed in experimental results. In our earlier research, we demonstrated the dependence of the effect on the particular conformation of the resorcylic ring coupled with the phenomenon of molecular aggregation. However, in light of the new results, it can be decisively concluded that the dual fluorescence effects observed in this group are intrinsically linked to the ESIPT process. It is, however, obvious that the dual fluorescence effect observed is strengthened by the molecular aggregation, which in turn suggests the involvement of AIE-related processes. Protonation of the thiadiazole ring in a low pH environment, as well as the emergence of anionic forms (the –O^−^ group) in high pH media, blocks the possibility of forming an intramolecular hydrogen bond and, therefore, the effect of dual emission. The previously described conformations “S” and “N” correspond to the *trans*-enol and *cis*-enol forms known from the literature, while the presence of the latter facilitates the formation of hydrogen bonds and emergence of ESIPT effects, which allows for the excited ketone tautomer to be formed in excited states, and consequently, the occurrence of long-wavelength fluorescence emission. It is also noteworthy that in the right conditions, we are able to force the effects related to dual fluorescence emission by triggering aggregation effects which may strengthen ESIPT processes. In our subsequent papers on this subject, we will focus on studying the effects that may emerge in the amorphic forms of these compounds, as well as further developing the theoretical [TD]DFT calculations.

## Figures and Tables

**Table 1:** Onsager cavity radius a_0_, slope m, change in dipole moment Δµ, coefficient of determination r^2^ and number of data for compound FABT and ADAPT.

Compound	a_0_[Å]	m_1_	Δµ [D]	r^2^	n
FABT	4.281	1866.541	2.098	0.79	9
		1313.541	1.760	0.92	7
		3548.132	2.893	0.98	4
ADAPT	4.829	234.435	0.891	0.86	7
		341.867	1.157	0.87	7

**Table 2:** Fluorescence lifetimes for ADAPT measured with the Time-Correlated Single Photon Counting (TCSPC) method after excitation with a 294 nm diode.

Buffer	Time [ns]
EtOH neutral	3.346 ± 0.004
EtOH basic	2.822 ± 0.004
EtOH acidic	1.26 ± 0.01
Chloroform	3.44 ± 0.02

**Table 3:** Fluorescence lifetimes for FABT measured with the TCSPC method after excitation with a 294 nm diode.

Buffer	Constituent	Lifetime [ns]	Fraction [%]
EtOH neutral	τ_1_	1.59 ± 0.054	(13.9 ± 1.2)
	τ_2_	0.794 ± 0.003	(86.1 ± 1.2)
EtOH basic	τ_1_	1.79 ± 0.01	(31.1 ± 0.4)
	τ_2_	0.22 ± 0.01	(68.9 ± 0.4)
EtOH acidic	τ_1_	5.0 ± 0.4	(1.8 ± 0.1)
	τ_2_	0.73 ± 0.1	(98.2 ± 0.1)
Chloroform	τ_1_	27.3 ± 0.2	(34.7 ± 0.1)
	τ_2_	1.67 ± 0.02	(25.4 ± 0.4)
	τ_3_	0.22 ± 0.01	(39.9 ± 0.3)

## Supplementary Materials

The following are available online. Scheme S1. Synthesis of ADAPT. Figure S1. 1H-NMR spectrum of ADAPT with inserts showing the numbering system of atoms and the expanded view of all signals. Figure S2. Fluorescence emission spectra for FABT in ethanol, DMF, chloroform and toluene. Figure 3S. Resonance light scattering spectra (RLS) for FABT (Panel A) and ADAPT (Panel B) in ethanol and chloroform. The RLS bands for FABT in ethanol were multiplied by the factor of 20 for better visibility of the effect. Figure S4. The infrared spectra for FABT in ethanol and in ethanol with the addition of acid or base. Figure S5. The Raman spectra for FABT in ethanol. Figure S6. The infrared spectra for ADAPT in ethanol and in ethanol with the addition of acid or base. Figure S7. The electronic absorption spectra for FABT (Panel A) and ADAPT (Panel B) in ethanol, as well as in ethanol after acidification to pH ~1 (apparent pH) and at pH ~10 after the addition of base. Figure S8. Fluorescence excitation spectra for FABT (Panel A) and ADAPT (Panel B) in ethanol and in ethanol with the addition of acid or base. Figure S9. Resonance light scattering spectra (RLS) for FABT (Panel A) and ADAPT (Panel B) in ethanol and in ethanol with the addition of acid or base. Figure S10. TCSPC analysis for FABT in EtOH and chloroform. The top panel presents the decay of fluorescence intensity. Black color denotes the apparatus profile. Points correspond to measurement results and the continuous line to the double and triple component analysis for measurements in ethanol and chloroform, respectively. The bottom panel presents the distribution of residuals. The excitation wavelength was 294 nm, fluorescence emission was recorded for wavelength >360 nm (emission filter F360). Figure S11. TCSPC analysis for ADAPT in EtOH and chloroform. The top panel presents the decay of fluorescence intensity. Black color denotes the apparatus profile. Points correspond to measurement results and the continuous line to the single component analysis. The bottom panel presents the distribution of residuals. The excitation wavelength was 294 nm, fluorescence emission was recorded for wavelength >360 nm (emission filter F360). Figure S12. Electronic absorption spectra of FABT (panel A) and ADAPT (panel B) in different solvent (chloroform and butan-1-ol) at different dilutions. Measurements were carried out for eight different concentrations C1–C8. The sample FABT and ADAPT (about 1 mg) were diluted in 1 mL of chloroform or butan-1-ol, and then appropriate aliquots of the solution were added to 3 mL of solvent. The chosen spectra with different concentration were shown in Figure S12. Figure S13. Fluorescence emission spectra of FABT (panel A,C) and ADAPT (panel B,D) according to Figure S14. Panel C shows the ratio between the fluorescence intensity from 487 nm to 405 nm according to different concentrations C1–C8. Figure S15. Electronic absorption spectra for FABT (panel A) and ADAPT (panel B) dissolved in H2O at varying pH (from 1.5 to 12). The spectra were not normalized. pH-metric titration curves for FABT (panel C) and ADAPT (panel D). Titration was carried out with the use of 1 M HCl. Figure S16. Fluorescence emission spectra of FABT (panel A) and ADAPT (panel B) dissolved in H_2_O at varying pH according to electronic absorption spectra. Table S1. Position of the maxima of absorption and fluorescence spectra for FABT and ADAPT in different solvents. Table S2. Dielectric constant ε, index of refraction n and solvent parameters F1(ε, n), F2(ε, n), ET(30), $E_{T}^{N}$ in different solvents. Table S3. Position of FTIR vibrations for FABT. The asterisk denotes both the solvent and molecule band. Table S4. Position of FTIR vibrations for ADAPT. The asterisk denotes both the solvent and molecule band. Table S5. Position of Raman shifts for FABT. The asterisk denotes both the solvent and molecule band.
