# Supplementary material for: ESIPT-Related Origin of Dual Fluorescence in the Selected Model 1,3,4-Thiadiazole Derivatives
Source: Molecules. 2020 Sep 11;25(18):4168. doi: 10.3390/molecules25184168 (PMC7570705; doi:10.3390/molecules25184168)
Supplement: Supplementary file 1 [file molecules-25-04168-s001.pdf]

ESIPT-Related Origin of Dual Fluorescence in the Selected Model 1,3,4-Thiadiazole Derivatives

Grzegorz Czernel [1], Iwona Budziak [2], Anna Oniszczuk [3,*], Dariusz Karcz [4,*], Katarzyna Pustuła [5], Andrzej Górecki [6], Alicja Matwijczuk [1], Bożena Gładyszewska [1], Mariusz Gagoś [7], Andrzej Niewiadomy [8] and Arkadiusz Matwijczuk [1,*]

[1] Department of Biophysics, University of Life Sciences in Lublin, Akademicka 13, 20-950 Lublin, Poland; grzegorz.czernel@up.lublin.pl (G.Z.); alicja.matwijczuk@up.lublin.pl (A.M.); bozena.gladyszewska@up.lublin.pl (B.G.)
[2] Department of Chemistry, University of Life Sciences in Lublin, Akademicka 15, 20-950 Lublin, Poland; iwona.budziak@up.lublin.pl
[3] Department of Inorganic Chemistry, Medical University in Lublin, 20-059 Lublin, Poland
[4] Department of Analytical Chemistry (C1), Faculty of Chemical Engineering and Technology, Cracow University of Technology, Warszawska 24, 31-155 Cracow, Poland
[5] Department of Theoretical Chemistry, Faculty of Chemistry, Jagiellonian University, Gronostajowa 2, 30-387 Kraków, Poland; pustulak@chemia.uj.edu.pl
[6] Department of Physical Biochemistry, Faculty of Biochemistry, Biophysics and Biotechnology of the Jagiellonian University, Gronostajowa 7, 30-387 Kraków, Poland; andrzej.gorecki@uj.edu.pl
[7] Department of Cell Biology, Institute of Biology and Biochemistry, Maria Curie-Skłodowska University, Akademicka 19, 20-033 Lublin, Poland; mariusz.gagos@poczta.umcs.lublin.pl
[8] Institute of Industrial Organic Chemistry, Annopol 6, 03-236 Warsaw, Poland; andrzej.niewiadomy@up.lublin.pl
* Correspondence: anna.oniszczuk@umlub.pl (A.O.); dariusz.karcz@pk.edu.pl (D.K.); arkadiusz.matwijczuk@up.lublin.pl (A.M.); Tel.: +48-814-456-937 (A.M.); Fax: +48-814-456-684 (A.M.)

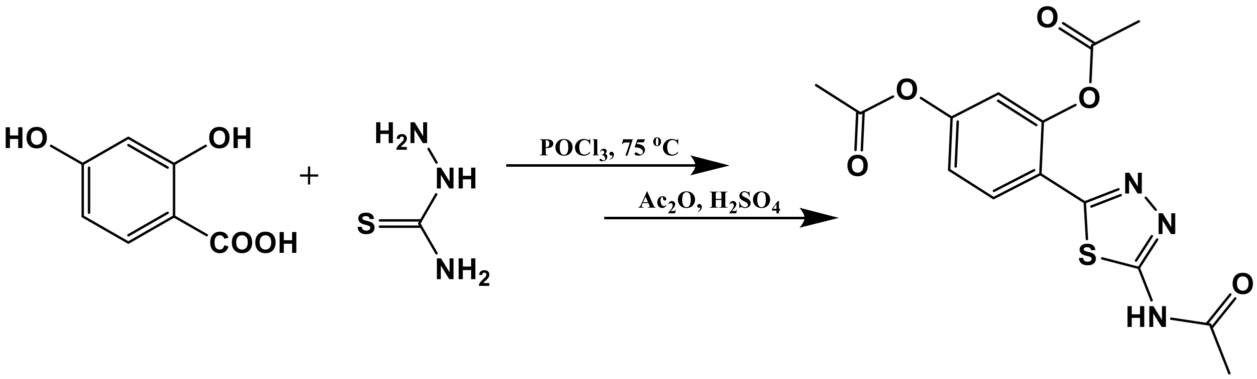

Scheme S1. Synthesis of ADAPT.

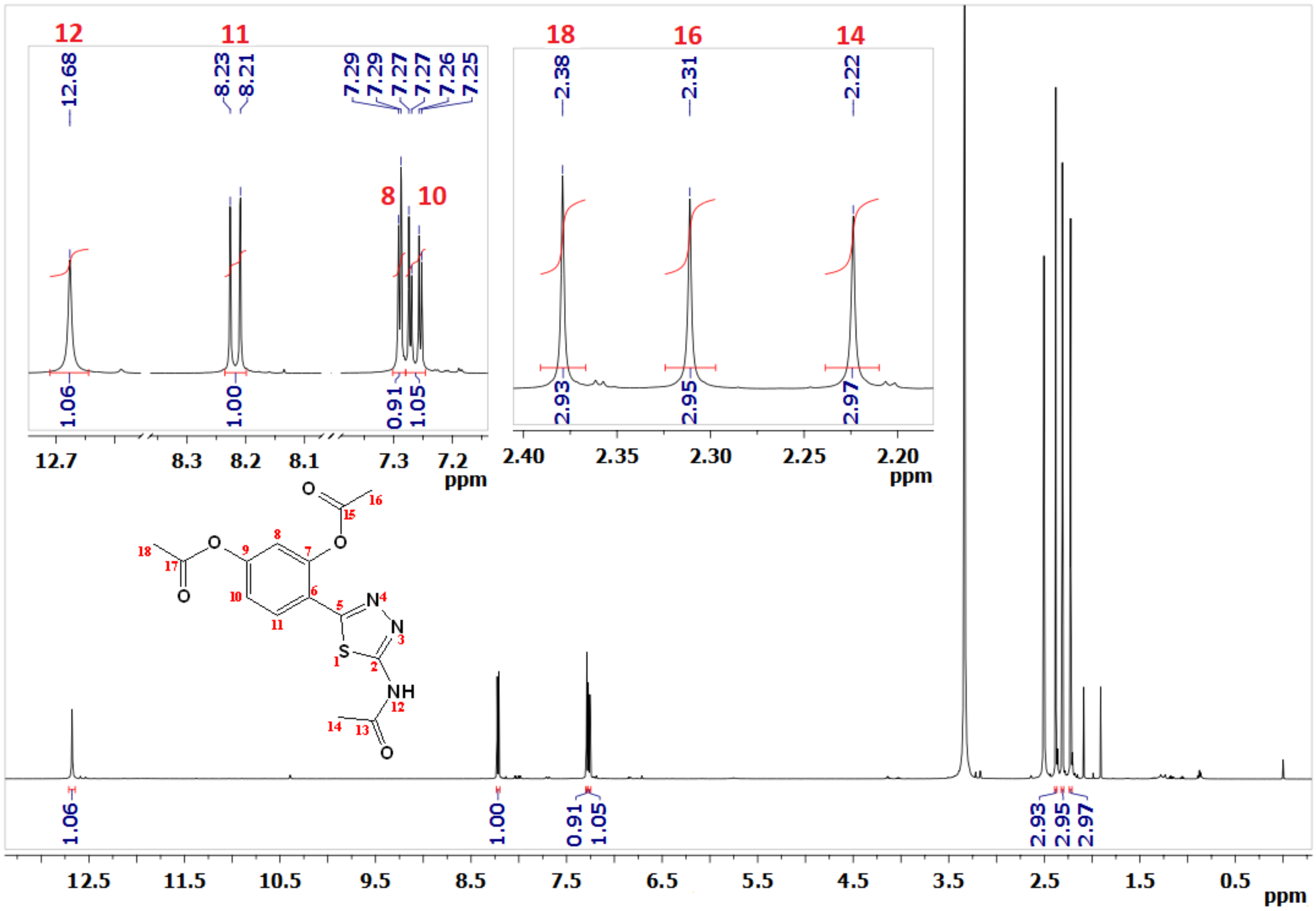

Figure S1. ^{1}H-NMR spectrum of ADAPT with inserts showing the numbering system of atoms and the expanded view of all signals.

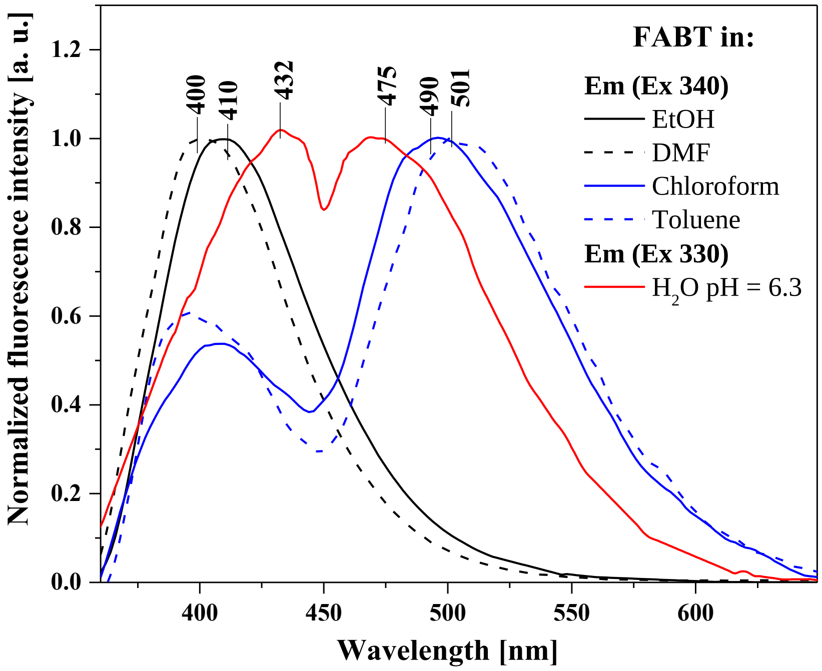

Figure S2. Fluorescence emission spectra for FABT in ethanol, DMF, chloroform and toluene.

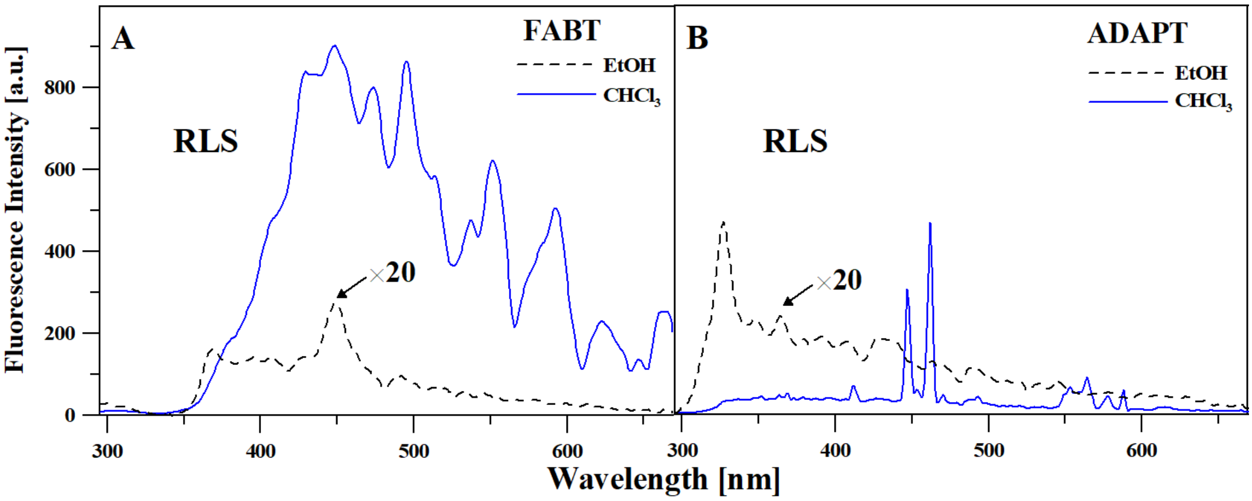

Figure S3. Resonance light scattering spectra (RLS) for FABT (Panel A) and ADAPT (Panel B) in ethanol and chloroform. The RLS bands for FABT in ethanol were multiplied by the factor of 20 for better visibility of the effect.

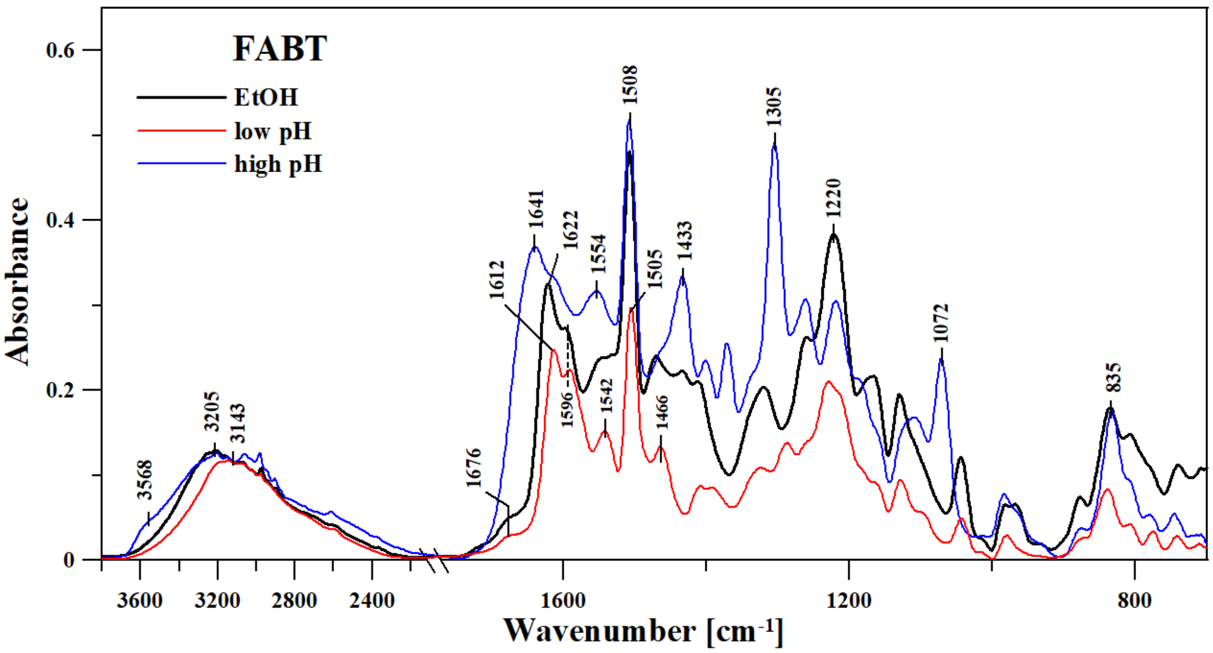

Figure S4. The infrared spectra for FABT in ethanol and in ethanol with the addition of acid or base.

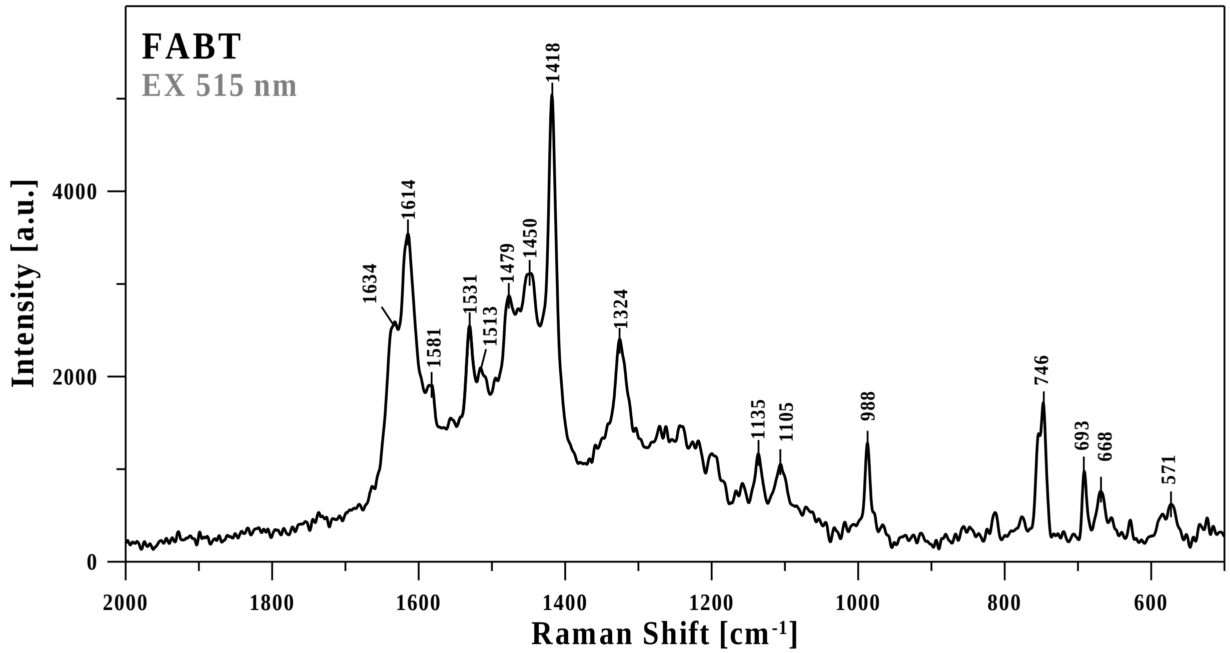

Figure S5. The Raman spectra for FABT in ethanol.

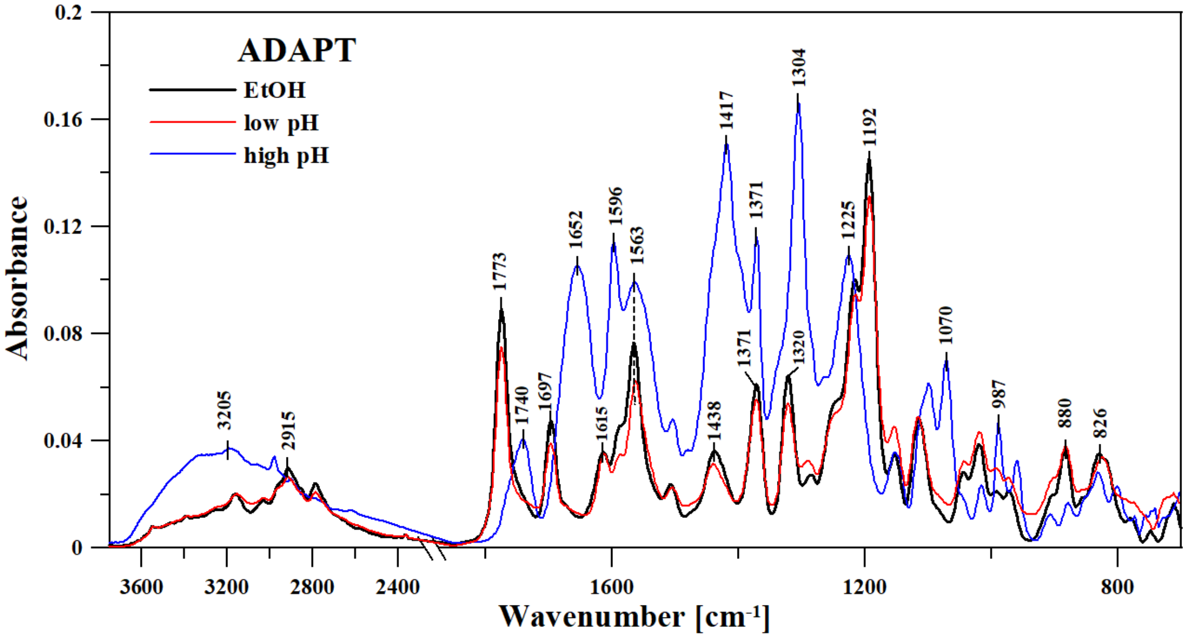

Figure S6. The infrared spectra for ADAPT in ethanol and in ethanol with the addition of acid or base.

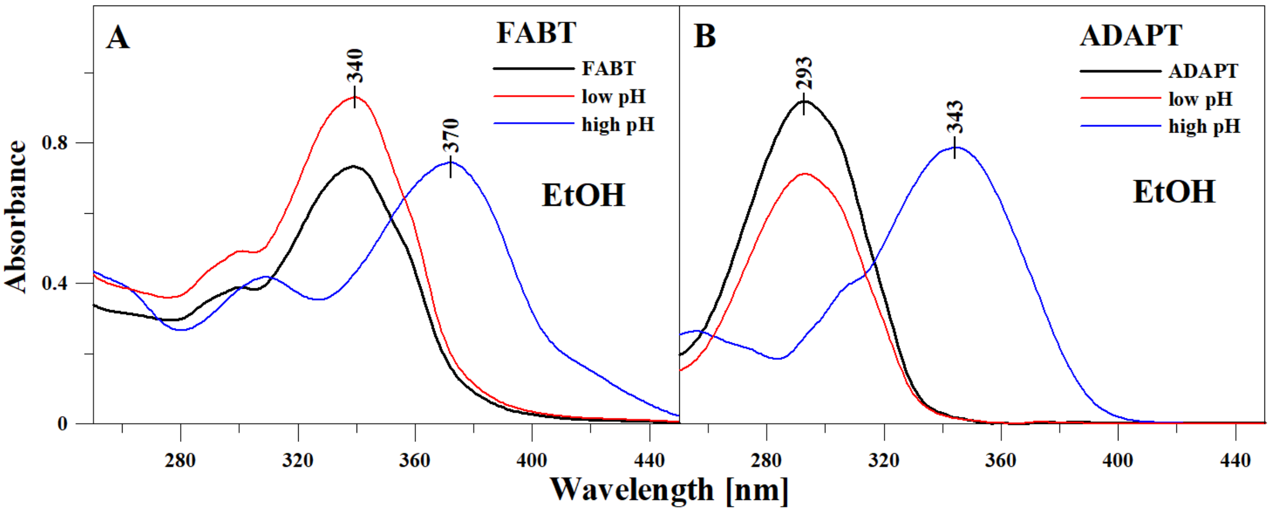

Figure S7. The electronic absorption spectra for FABT (Panel A) and ADAPT (Panel B) in ethanol, as well as in ethanol after acidification to pH ~ 1 (apparent pH) and at pH ~10 after the addition of base.

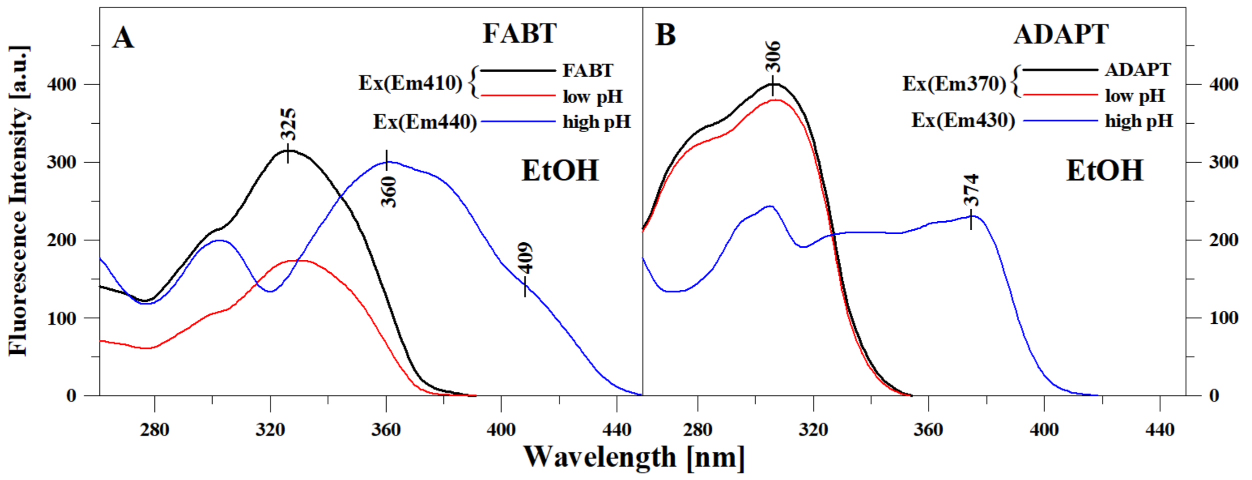

Figure S8. Fluorescence excitation spectra for FABT (Panel A) and ADAPT (Panel B) in ethanol and in ethanol with the addition of acid or base.

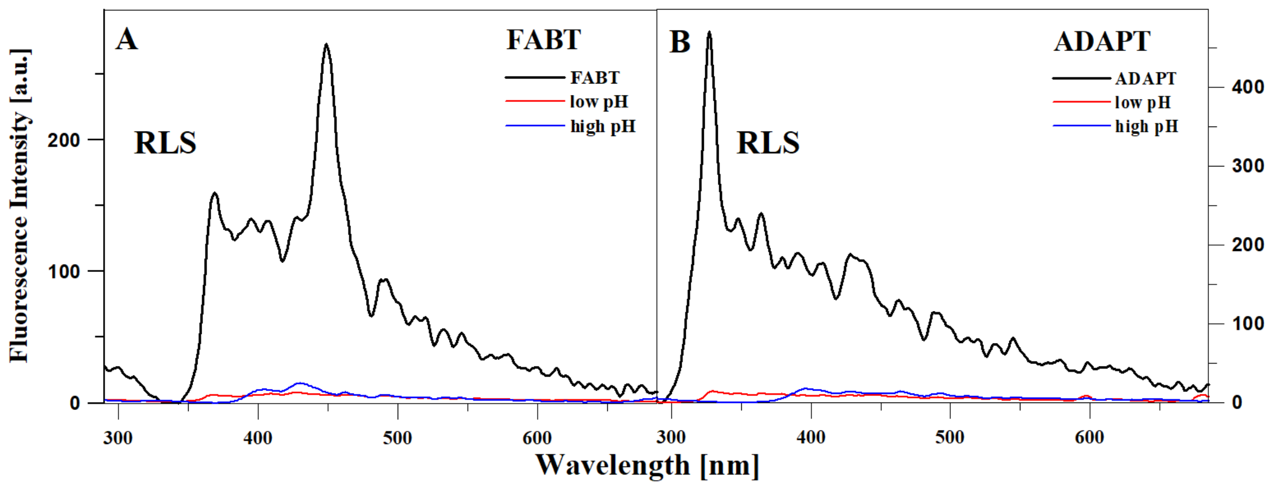

Figure S9. Resonance light scattering spectra (RLS) for FABT (Panel A) and ADAPT (Panel B)) in ethanol and in ethanol with the addition of acid or base.

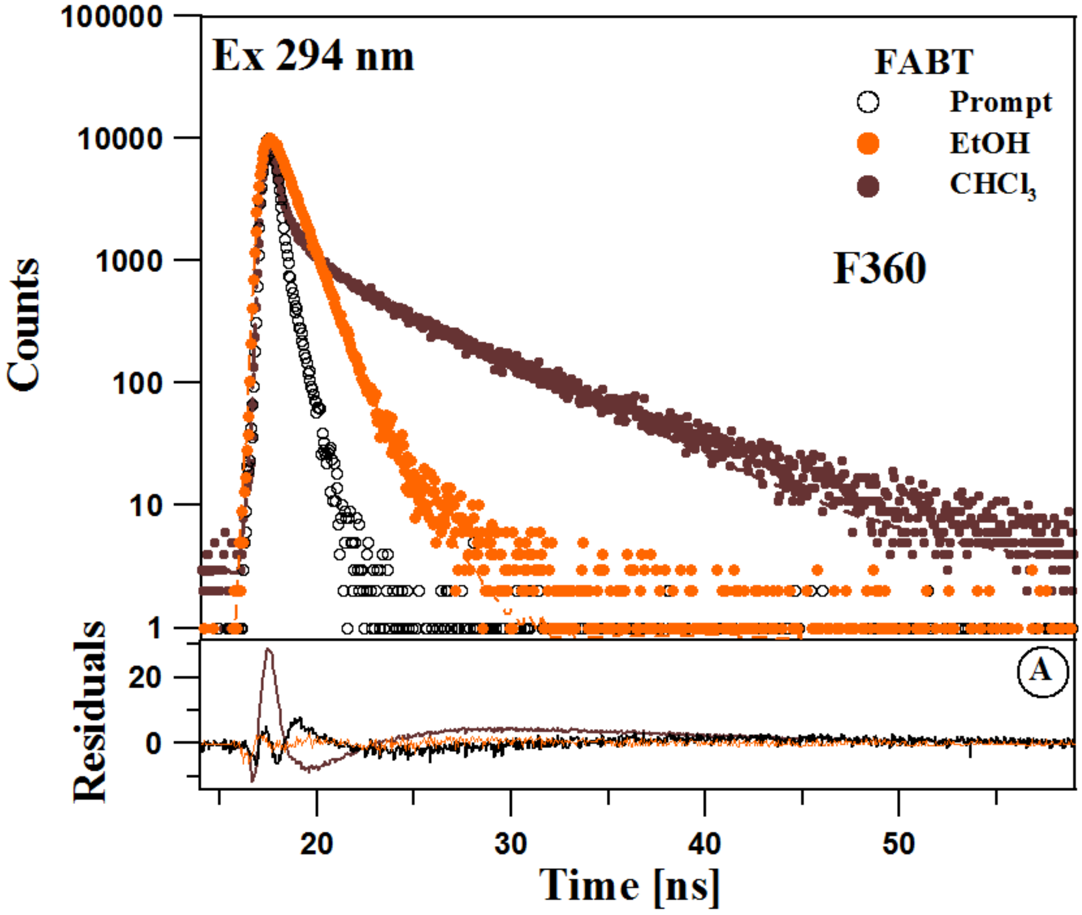

Figure S10. TCSPC analysis for FABT in EtOH and chloroform. The top panel presents the decay of fluorescence intensity. Black color denotes the apparatus profile. Points correspond to measurement results and the continuous line to the double component analysis. The bottom panel presents the distribution of residuals. The excitation wavelength was 294 nm.

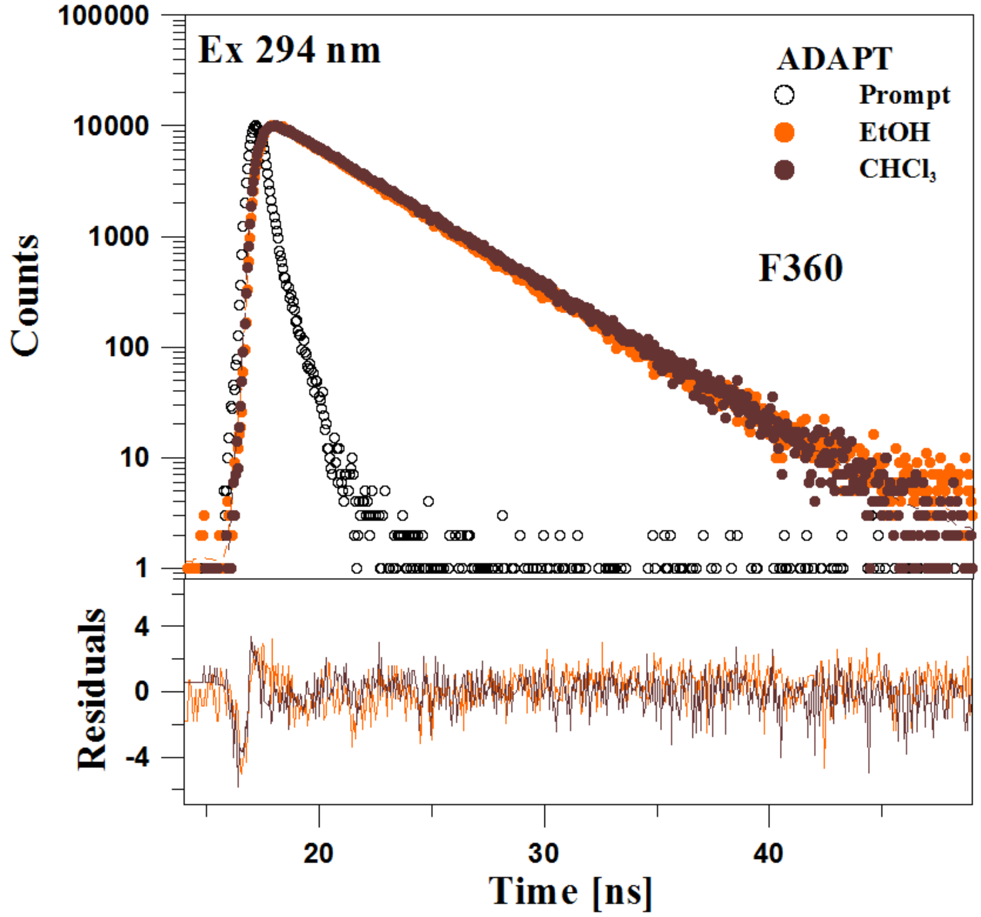

Figure S11. TCSPC analysis for ADAPT in EtOH and chloroform. The top panel presents the decay of fluorescence intensity. Black color denotes the apparatus profile. Points correspond to measurement results and the continuous line to the double component analysis. The bottom panel presents the distribution of residuals. The excitation wavelength was 294 nm.

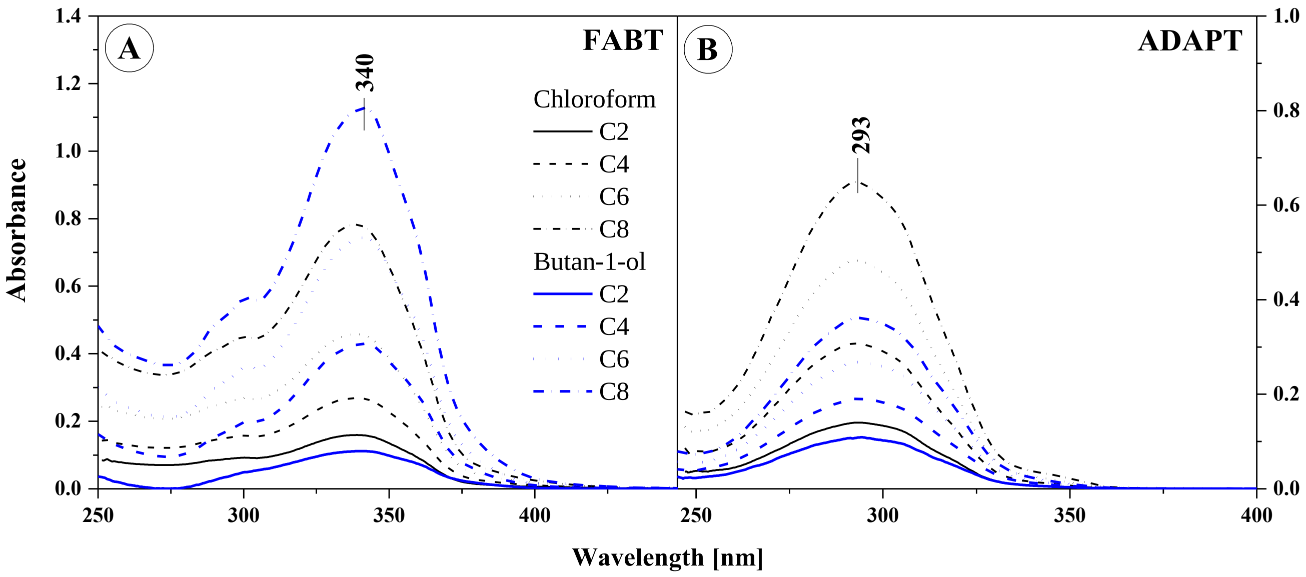

Figure S12. Electronic absorption spectra of FABT (panel A) and ADAPT (panel B) in different solvent (chloroform and butan-1-ol) at different dilutions. Measurements were carried out for eight different concentration C1-C8. The sample FABT and ADAPT (about 1 mg) were diluted in 1 ml of chloroform or butan-1-ol, and then appropriate aliquots of the solution were added to 3 mL of solvent. At the figure 1 chosen spectra with different concentration were shown.

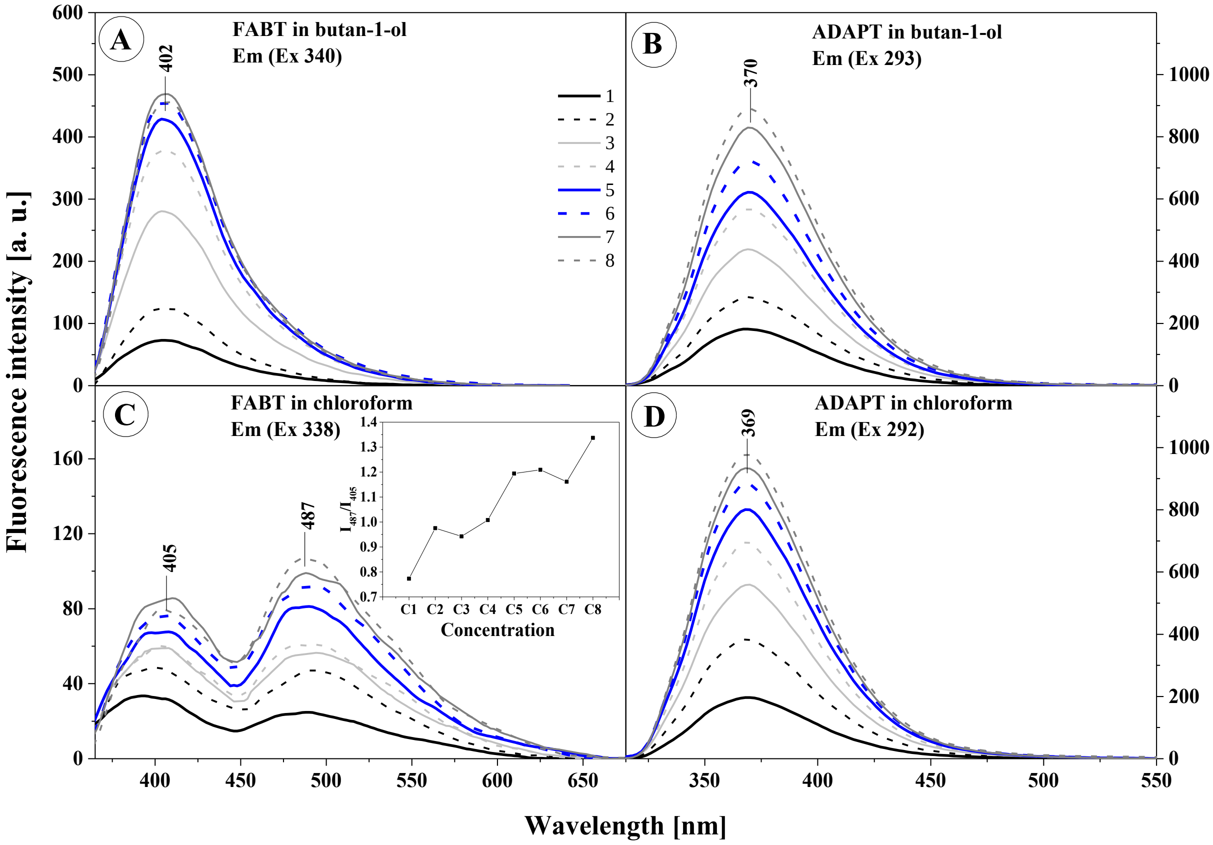

Figure S13. Fluorescence emission spectra of FABT (panel A and C) and ADAPT (panel B and D) according to fig. S12. Panel C shows the ratio between the fluorescence intensity at 487 nm to 405 nm according to different concentration C1-C8.

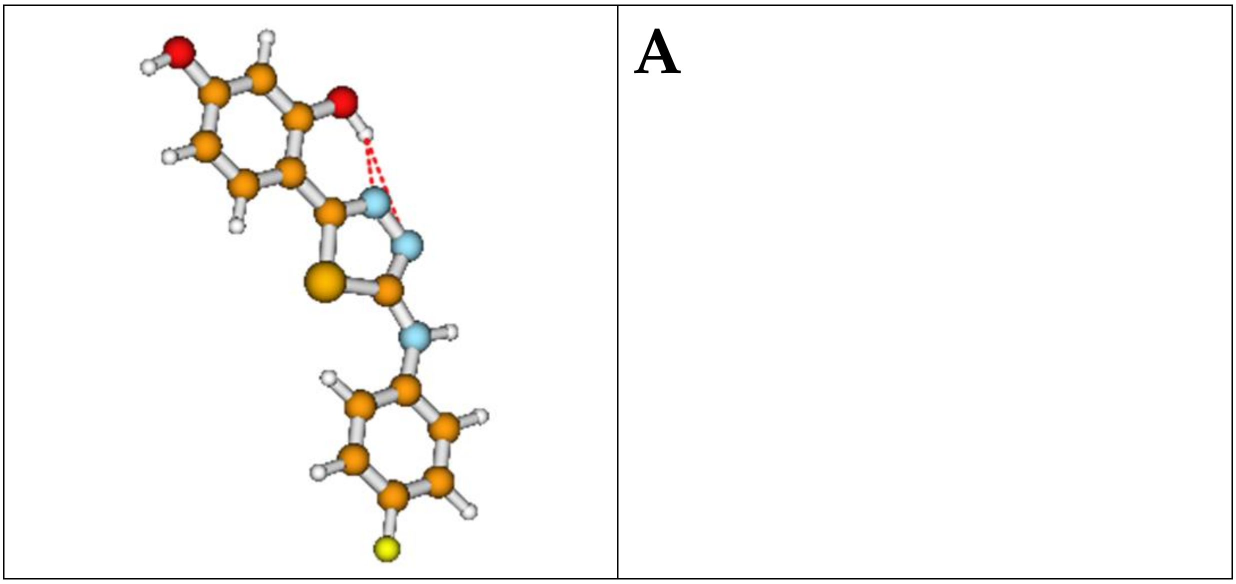

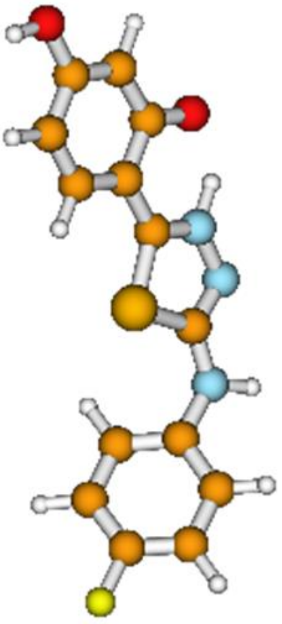	**B**
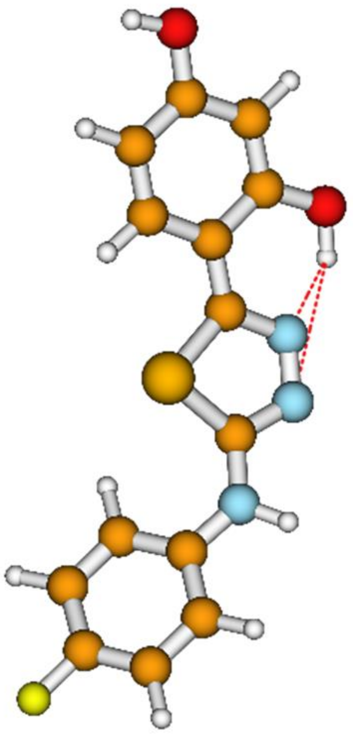	**C**

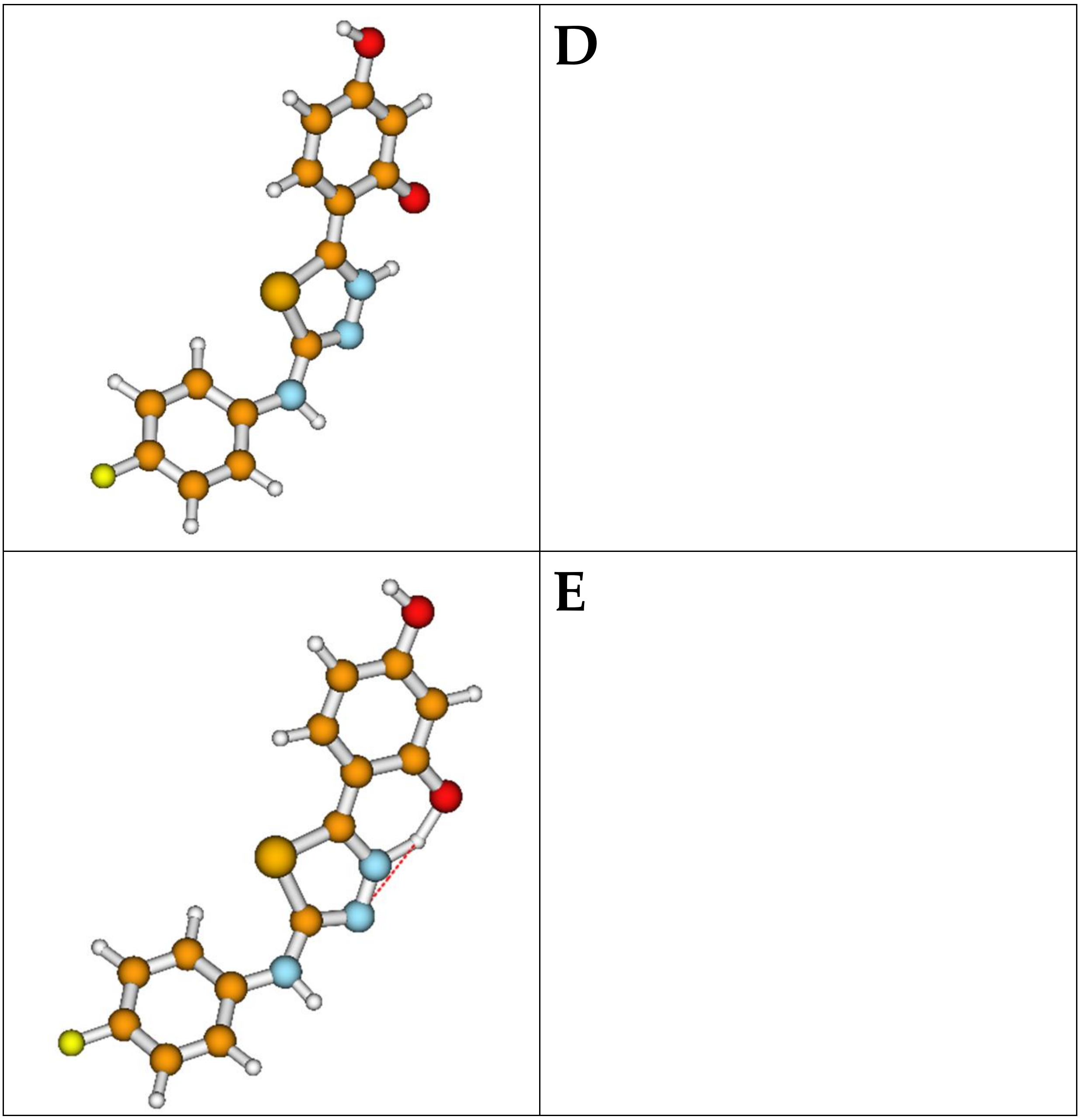

Figure S14. Panels A and B: Optimized structures of the enol and keto forms of **FABT**. Panels C, D, and E – respective excited sate geometric forms for the enol, keto, and transition state (TS).

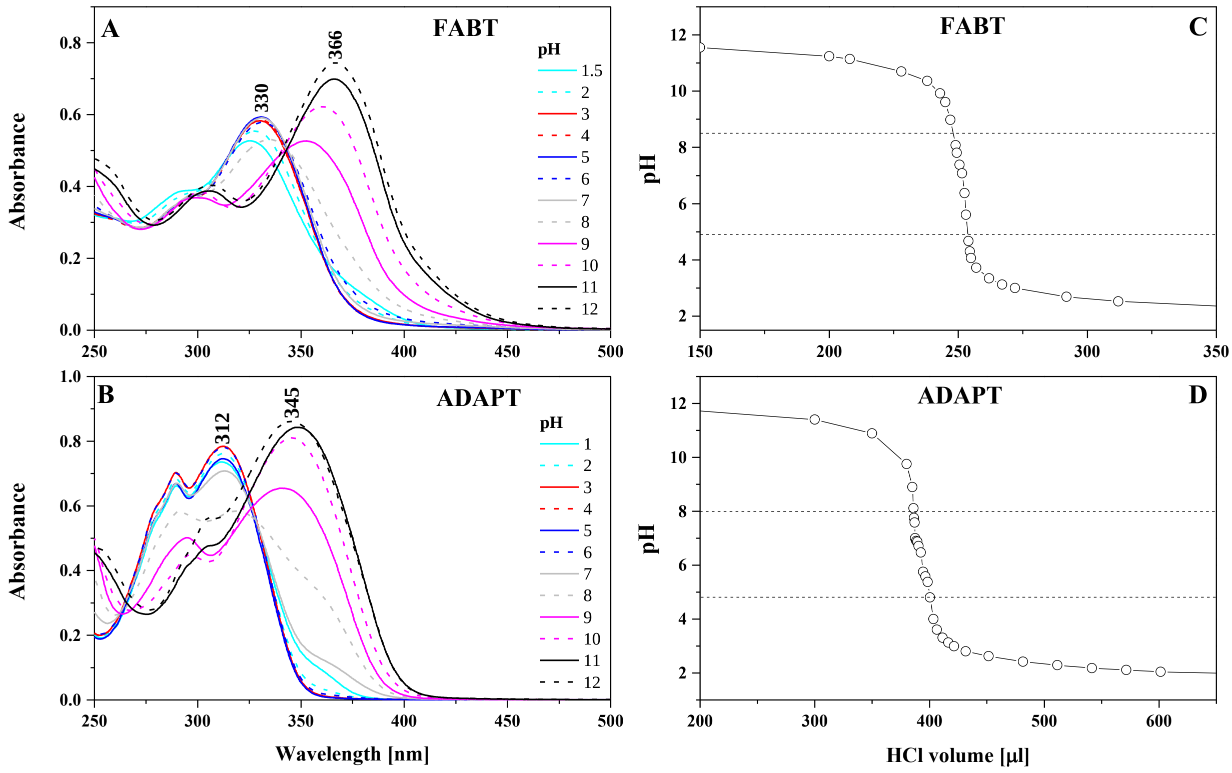

Figure S15. Electronic absorption spectra for FABT (panel A) and ADAPT (panel B) dissolved in H_2O at varying pH (from 1.5 to 12).The spectra were not normalized. pH-metric titration curves for FABT (panel C) and ADAPT (panel D). Titration was carried out with the use of 1 M HCl.

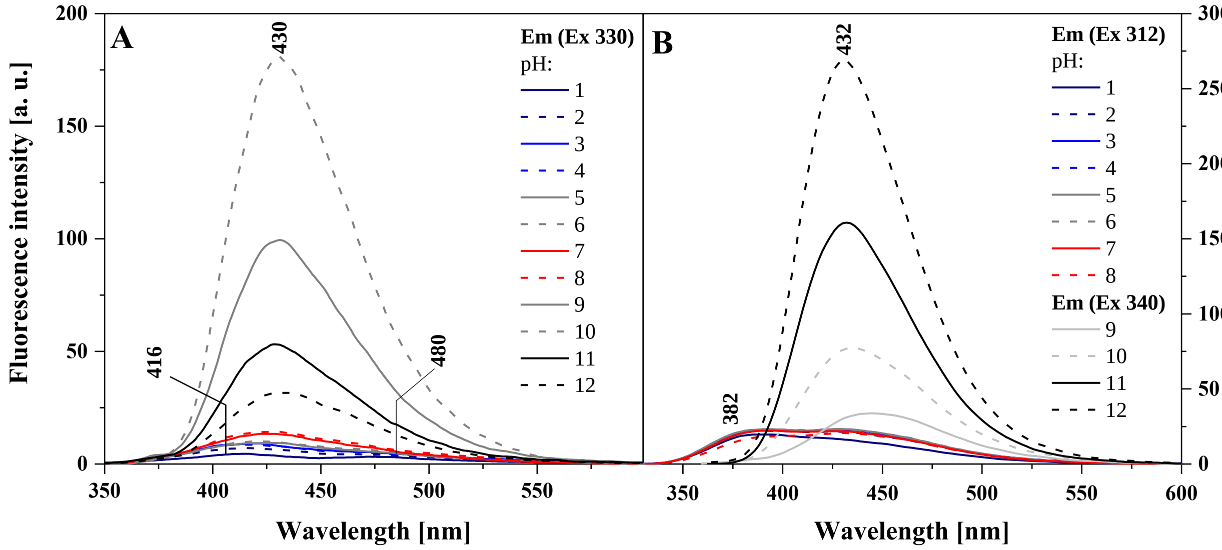

Figure S16. Fluorescence emission spectra of FABT (panel A) and ADAPT (panel B) dissolved in H_2O at varying pH according to electronic absorption spectra.

Table S1. Position of the maxima of absorption and fluorescence spectra for FABT and ADAPT in different solvents.

Solvent	FABT Absorbance [nm]/[cm^{-1}]		FABT Fluorescence [nm]/[cm^{-1}]		ADAPT Absorbance [nm]/[cm^{-1}]		ADAPT Fluorescence [nm]/[cm^{-1}]	
Methanol	336	29762	414	24155	292	34247	371	26954
Butan-1-ol	342	29240	412	24272	294	34014	373	26810
Ethanol	337	29674	412	24272	293	34130	372	26882
Propan-2-ol	340	29412	407	24570	293	34130	372	26882
Acetonitrile	337	29674	407	24570	291	34364	370	27027
Ethyl acetate	340	29412	399	25063	292	34247	369	27100
DMSO	340	29412	409	24450	296	33784	376	26596
DMF	340	29412	408	24510	297	33670	380	26316
Acetone	340	29412	404	24752	-	-	-	-
Toluene	340	29412	406	24631	293	34130	372	26882
			501	19960				
Cyclohexane	342	29240	404	24752	293	34130	380	26316
			502	19920				
Chloroform	340	29412	414	24155	293	34130	371	26954
			500	20000				
THF	340	29412	412	24272	294	34014	371	26954

Table S2. Dielectric constant ε, index of refraction n and solvent parameters $E_T(30)$, E_T^N in different solvents.

Solvent	ε	n	$E_T(30)$	E_T^N

Methanol	33	1.3265	55.5	0.765
Butan-1-ol	17.8	1.3993	49.7	0.586
Ethanol	25.3	1.3594	51.9	0.654
Propan-2-ol	20.18	1.3772	48.6	0.552
Acetonitrile	36.64	1.3416	45.6	0.460
Ethyl acetate	6.02	1.3723	38.1	0.228
DMSO	47.24	1.4773	45.1	0.444
DMF	36.7	1.4305	43.2	0.386
THF	7.58	1.4072	37.4	0.207
Acetone	21.0	1.3587	42.2	0.355
Toluene	2.38	1.4969	33.9	0.099
Cyklohexane	2.02	1.4262	30.9	0.006
Chloroform	4.81	1.4429	39.1	0.259

Table S3. Position of FTIR vibrations for FABT. The asterisk denotes both the solvent and molecule band.

**FTIR - band position [cm^{-1}]**			**Vibration**
**EtOH**	**EtOH + HCl**	**EtOH +NaOH**	
3205	3143	3568 / 3205	ν (O-H)
1676	1676		ν (C=O) and ν (C=N)
1622	1612	1641 / 1612	ν (C=N)
1596	1596		
1546	1542	1554	
1508	1505	1508	
1433* / 1466*	1466*	1433*	δ (C-H) + ν (C=C)
1410	1410		
		1402	δ (C-H) + ν (C=C)
	1390		
		1371	
1321	1321		ν (C-N) in C=N-C δ (C-H)
		1305	or ν (C-O)
	1287		δ (N-H) + ν (C-N)
1261		1261	

	1230		
1220		1220	
1166			δ (C-C) in C-(C=O)-C
1128*	1128*		
		1111*	ν (C-N)
		1072	ν (C-O-C)
1043	1043		
980	980	980	ν_{as}(C-N) oraz δ (C-H)
966			
876	876	878	
835	835	835	

ν - valence vibration; δ – deformation; s - symmetric; as – asymmetric

Table S4. Position of FTIR vibrations for ADAPT. The asterisk denotes both the solvent and molecule band.

**FTIR - band position [cm^{-1}]**			**Vibration**
**EtOH**	**EtOH + HCl**	**EtOH + NaOH**	
		3205	ν (O-H) in NaOH
2915	2915		ν (N-H)
1773		1773 / 1740	ν (C=O)
1697	1697	1652	ν (C=O) and ν (C=N)
1615	1615		ν (C=N)
		1596	
1563	1563	1563	
1438*	1438*	1417	δ (C-H) + ν (C=C)
1371	1371	1371	δ (C-H) + ν (C=C)
1320	1320		δ (N-H) + ν (C-N)
		1304	
		1225	
1192	1192		δ (C-C) in C-(C=O)-C and ν (C-N)
1114*	1114*		
		1097	ν (C-O-C)
		1070	
987	987	987	ν_{as}(C-N) oraz δ (C-H)
880	880	880	
876	876	876	
835	835	835	

ν - valence vibration; δ – deformation; s - symmetric; as – asymmetric

Table S5. Position of Raman shifts for FABT. The asterisk denotes both the solvent and molecule band.

**RAMAN** FABT in EtOH	Vibration
1634	ν(C=N)
1614 / 1581	
1531	ν(C=C)
1513	
1479	
1450*	δ(C-H) + ν(C=C)
1418	δ(C-N) in C=N-C and δ(C-H)
1324	
1135	ν(C-O) or δ(N-H) + ν(C-N)
1105*	ν(C-N)
988	ν(C-F)
746	ν(C-F) and solvent
693 / 668 / 571	

Table S6. The plane under the emission curve, the value of absorbance at the excitation wavelength and fluorescence quantum yield for FABT and ADAPT.

	**FABT**			**ADAPT**		
Solvent	I^a	λ_{ex}	Φ_F	I^a	λ_{ex}	Φ_F
Methanol	4757.9354	317	0.0591	6569.5102	272	0.0948
Ethanol	5461.8577	321	0.0703	6700.7614	272	0.1019
Chloroform	40.4381	320	0.0006	4703.9238	272	0.0804
Toluene	37.3047	320	0.0006	25.3899	273	0.0005

[a] the plane under the emission curve

Table S7. Vertical excitation energies (E), oscillator strength (f), and juxtaposition with experimental data.

**E (eV)**	**f**	**Maxima of experimental bands (eV)**
3.50	0.86	3.68 (strong)
4.13	0.02	4.14 (slight enhancement of an intensive band)

Table S8. Vertical fluorescence energies (E), their origin and juxtaposition with experimental data.

E (eV)	Origin	Maxima of experimental fluorescence bands (eV)
2.66	Keto form	2.50
2.75	Enol form	2.92

Table S9. Energy (in kcal/mol) of various electronic states relative to the minimum in the enol ground state.

Name	E [kcal/mol]
Enol ground state	0
Enol – excited state energy minimum	71.02
Enol – excited state in the ground state geometry	80.71
Ketone	7.52
Ketone – excited state energy minimum	73.14
TS for the excited state enol->ketone transfer	77.20
Excitation light energy (328 nm)	87.16
